# Lightsheet localization microscopy enables fast, large-scale, and three-dimensional super-resolution imaging

**DOI:** 10.1038/s42003-019-0403-9

**Published:** 2019-05-09

**Authors:** Chieh-Han Lu, Wei-Chun Tang, Yen-Ting Liu, Shu-Wei Chang, Frances Camille M. Wu, Chin-Yi Chen, Yun-Chi Tsai, Shun-Min Yang, Chiung-Wen Kuo, Yasushi Okada, Yeu-Kuang Hwu, Peilin Chen, Bi-Chang Chen

**Affiliations:** 1 Research Center for Applied Sciences, Academia Sinica, Taipei, 11529 Taiwan; 2 Institute of Physics, Academia Sinica, Taipei, 11529 Taiwan; 3 Laboratory for Cell Polarity Regulation, Center for Biosystems Dynamics Research, RIKEN, Suita, Osaka, 565-0874 Japan; 4 Department of Physics, Universal Biology Institute and International Research Center for Neurointelligence, The University of Tokyo, Tokyo, 113-0033 Japan; 5 Brain Research Center, National Tsing Hua University, Hsinchu, 30013 Taiwan

**Keywords:** Super-resolution microscopy, Nuclear envelope, Super-resolution microscopy, Nuclear envelope

## Abstract

Recent advances in super-resolution microscopy allow the localization of single molecules within individual cells but not within multiple whole cells due to weak signals from single molecules and slow acquisition process for point accumulation to reconstruct super-resolution images. Here, we report a fast, large-scale, and three-dimensional super-resolution fluorescence microscope based on single-wavelength Bessel lightsheet to selectively illuminate spontaneous blinking fluorophores tagged to the proteins of interest in space. Critical parameters such as labeling density, excitation power, and exposure time were systematically optimized resulting in a maximum imaging speed of 2.7 × 10^4^ µm^3^ s^−1^. Fourier ring correlation analysis revealed a reconstructed image with a lateral resolution of ~75 nm through the accumulation of 250 image volumes on immobilized samples within 15 min. Hence, the designed system could open new insights into the discovery of complex biological structures and live 3D localization imaging.

## Abstract (web summary)

Lu, Tang, Liu et al. present a 3D super-resolution fluorescence microscope that uses selective plane illumination and a spontaneously blinking dye for localization-based imaging. They show that their microscope can be used to study complex biological structures.

## Introduction

Quantitative measurements and integrated analysis of structural and molecular dynamics are necessary to visualize the unseen complex and dynamic biological structures present in living cellular organisms^1–3^. Conventional fluorescence microscopy is one of the most broadly used imaging techniques in cell biology. It enables straightforward observations of cellular structures and biomolecular dynamics by labeling molecules with fluorophores^4^ or fluorescent proteins^5^ at the single-molecule level. For instance, movements of motor proteins such as ATPase^6^ or actomyosin^7^ have been recorded with an accuracy of few nanometers. However, the inherent nature of fluorescence microscopy slows its further increase in resolving power for the differentiation of the behavior of several biomolecules within a diffraction-limited area.

To circumvent the barriers imposed by the diffraction limit, several super-resolution techniques have been demonstrated in various biological systems. These techniques include stimulated emission depletion microscopy^8^, structured illumination microscopy^9^, photo-activated localization microscopy (PALM)^10^, and stochastic optical reconstruction microscopy (STORM)^11^. The last two, PALM and STORM, evolved naturally from single-molecule spectroscopy where the precise location of the single molecules could be determined by localization-based imaging. One crucial criterion for the aforementioned localization-based microscopy is that fluorescence signals should only originate from a single molecule within the diffraction-limited area during the acquisition period^12,13^. Despite the fact that localization-based super-resolution microscopy has been extensively used for studies that yield biological impacts^14–16^, several disadvantages are yet to be solved, such as the use of high power laser sources, challenges in obtaining a clear 3D image in thick samples, long data acquisition times, and limited field-of-view.

In a typical PALM or STORM setup, photon-activated fluorophores are generally excited by two lasers: one for activation and the other for inactivation of all fluorophores. Debut of direct stochastic optical reconstruction microscopy (dSTORM)^17^ merged this two-step photo-activation process into one; later, ground state depletion microscopy followed by individual molecule return (GSDIM)^18^ was introduced for controlled chemical environments to ensure that the fluorophores remain in the dark state for a sufficiently long time before they stochastically return to the ground state. Nevertheless, using a high laser power density to deplete the fluorophores in the ground state could induce strong photobleaching and consequently damage the biological samples restricting in vivo applications.

To address these concerns, the spontaneously blinking dye, HMSiR, was introduced for super-resolution microscopy^19^ in which the fluorophores in their native states are non-fluorescent. The HMSiR blinking fluorophore is a rhodamine derivative bearing an intramolecular nucleophile that is in thermal equilibrium between its fluorescent and non-fluorescent forms. The on-state and off-state interconvert via an intramolecular spirocyclization reaction resulting in a spontaneously blinking activity in the ground state^19^. These HMSiR blinking fluorophores were converted to on-state stochastically by thermal fluctuation, which can then be imaged via a single-wavelength laser with relatively low power intensity.

In conventional localization-based super-resolution microscopy, the system is limited to 2D images because axially overlapped fluorophores cannot be separated. By introducing optical astigmatism, axial positions can be determined in a thin sample expanding the dimension to super-resolved 3D images^20^. However, for thick samples, out-of-focus fluorescence signals contribute to the noise and overlapping of fluorescence signals in the axial position, which makes it nearly impossible to obtain high-quality 3D super-resolution images. This predicament could be solved by employing optical sectioning techniques, such as temporal focusing^21^, multi-focus grating^22^, and selective plane illumination microscopy (SPIM)^23–25^. Of these, SPIM separates the excitation axis from the detection axis, and thus it has inherent optical sectioning capability by illuminating an ultra-thin excitation plane (roughly half a micron) that is orthogonal to the detection axis. In this configuration, only fluorophores within this ultra-thin excitation plane can be excited implicitly eliminating most out-of-focus fluorescence background signals and reducing the unwanted photobleaching^25,26^.

Several research breakthroughs in super-resolution imaging techniques on single-molecule localization were achieved through standard fluorescent molecules or dyes, such as fluorescence-PALM^27^, bleaching or blinking-assisted localization microscopy^28,29^, and applications of lipophilic cyanine dyes^30,31^; however, their applications in live 3D imaging^32,33^ has remained limited due to associated phototoxicity and photodamage effects.

Here, lightsheet localization microscope was designed based on a scanning Bessel beam and spontaneously blinking HMSiR fluorophores and demonstrated using super-resolved 3D images of microtubules and nuclear pore structures in fixed or live samples. In addition, the red single-wavelength laser (*λ* = 637 nm) used to excite HMSiR fluorophores reduced the phototoxicity imposed on the live cells. Moreover, the imaging volume was extended by the sample-scan configuration providing a vast imaging area within a single scan. This gentle and rapid large-scale localization-based imaging system could be beneficial for live 3D localization.

## Results

### Design of a large-scale localization-based imaging system

To build a microscope that can perform localization-based imaging over a large volume of interest, two primary requirements must be fulfilled: reduced out-of-focus fluorescence background and an imaging mechanism feasible for large volumes. As mentioned earlier, SPIM is a promising candidate microscope system that satisfies these requirements because of its inherent optical sectioning capability, and its ability to image large samples. Gaussian beam lightsheet illumination has been widely used in conventional selective plane illumination microscopy; however, the thickness of a traditional lightsheet is often thicker than the depth-of-view of the high numerical-aperture objective used in localization microscopy—especially when the length of the lightsheet must cover a large imaging volume. Thus, a Bessel beam plane illumination was introduced to extend the lightsheet coverage while maintaining the optical sectioning^34^. The non-diffracting characteristics of a Bessel beam allow it to create a thinner lightsheet versus the same length of the Gaussian beam. This leads to better axial resolution. The thickness of the scanning-Bessel beam lightsheet can be tuned to match the depth-of-view of the detection objective, which minimizes the out-of-focus fluorescence background over a long propagation distance.

There are two scanning strategies adapted in this localization-based microscope system to cover a large imaging volume: sample scan and objective scan. In the sample scan, the sample is placed on a motorized sample holder and moved across the virtual lightsheet illumination plane^35^. In an objective scan, the illumination lightsheet is swept across the sample by a scanning mirror synchronized with the detection objective mounted on a piezo stage^35,36^. In the second scanning scheme, synchronization between lightsheet illumination and detection objective is necessary to ensure precise identification of fluorescent molecules. However, for observations that require a large field-of-view, it is challenging to coordinate the illumination plane and detection objective due to optical alignment and aberrations. Moreover, to cover a large imaging volume, a long and thick lightsheet is needed for good signal-to-noise, which leads to a poor axial resolution. For localization imaging based on objective scans, the inconsistency of the detection plane and the illumination plane will reduce the fluorescence intensity and alter the point spread function (PSF). Detailed discussions on this issue can be found in the Supplementary Information and Supplementary Fig. 1.

**Fig. 1:**
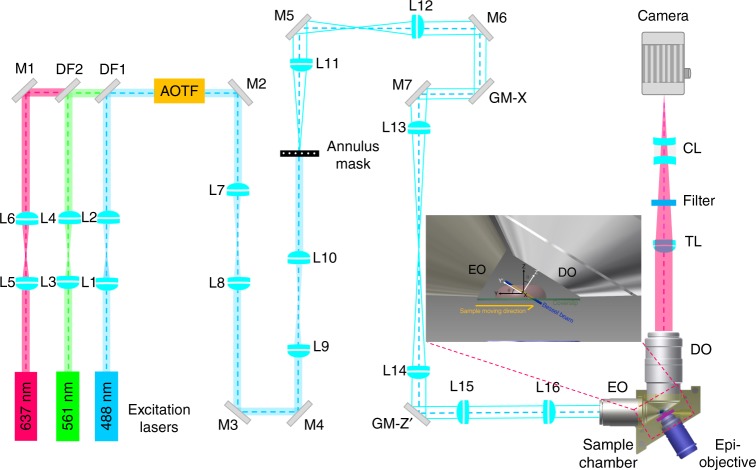
Schematic representation of selective plane illumination microscope based on Bessel beam illumination. The inset shows a detailed sample-scanning configuration between the thin excitation plane and the sample specimen. L lens, M mirror, DF dichroic filter, AOTF acousto-optical tunable filter, GM-*X* galvanometer mirror in *x*, GM-*Z*′ galvanometer mirror in *z*′, EO excitation objective, DO detection objective, TL tube lens, CL cylindrical lens. *Note*: This schematic diagram is not drawn into scale. The black coordinate system (*XYZ*) indicates the sample orientation while the white coordinate system (*X*′*Y*′*Z*′) represents axes used in images taken by objective scan where *X*-axis overlaps with *X*′-axis

Therefore, a sample scan strategy was employed in our system because maintaining a stationary illumination lightsheet could ensure that the system is spatially invariant over a large scanning area. The sample is scanned step-wise via the closed-loop piezo linear stage and repeatedly swept through the illumination plane during the imaging process (see Fig. 1 and Supplementary Fig. 2). Since the detection plane was fixed in the microscope system, the spatial variation of the overall PSF and the associated defocused calibration curves (see Supplementary Fig. 3) were minimized. More importantly, the imaging volume is no longer limited by the field-of-view of detection optics but rather defined by the automatic sample stage controller. Here, the developed microscope system can successfully cover volumes from 10^4^ to 10^5^ μm^3^ at a speed of 2–3 s per volume depending on the exposure time and step size.

Importantly, the imaging volume of the designed large-scale localization-based system increased versus conventional localization microscope techniques. In conventional localization-based imaging techniques, the intense excitation laser is generally transmitted through the entire sample when using the epi-illumination approach. This leads to severe photobleaching and photodamage effects when one attempts to acquire a large-scale super-resolution image of a thick sample. Bessel beam plane illumination does not restrict the imaging area to the closed vicinity of the coverslip surface. It is now possible to observe samples with structures that protrude from the basal side of the coverslip, such as 3D-cultured cells or tissues that are not feasible in conventional localization-based imaging techniques.

To increase the imaging speed while maintaining a good resolution, one crucial factor is to find a balance between localization precision and image acquisition time. The relationship between the fitting uncertainty and exposure time, laser power, and number of detected photons was systematically investigated and evaluated for analysis (see Supplementary Figs. 4 and 5). The total number of localized fluorophores reached a maximum value at a relatively low power density and decreased with increasing laser power. Although higher excitation power could increase the number of detected photons, it could also reduce the number of localized fluorophores because of photobleaching and increased out-of-focus fluorescence background. Hence, we optimized the excitation power density at 0.17–0.33 kW cm^−2^ (see Supplementary Fig. 5c, d). Moreover, a similar relationship was found between the exposure time and the theoretical localization uncertainty; exposure time was found to be optimal for efficient image acquisition around 50–75 ms under current sample preparation procedures (see Supplementary Fig. 5e, f).

### 3D Large-scale imaging of intracellular fine structures

In conventional super-resolution optical microscopy, a high-resolution image relies on the reconstruction of thousands of frames and a longer acquisition time resulting in associated photobleaching and phototoxicity effects. Moreover, long-term imaging typically results in poor image quality due to fluctuations in the surrounding environment, thermal instability, and deformation of the sample specimen^24^. Therefore, to reduce the imaging time for a super-resolution image, the labeling density was increased such that 1.7 fluorophores can be registered per µm^2^ frame^−1^ on the average—this is close to the high density of fluorophores defined in the localization microscopy data processing^24,37^. This strategy is not applicable in conventional localization-based technique because a high density of fluorophores could induce a notably high fluorescence background due to epi-illumination that reduces localization precision. An ultra-thin plane illumination by the scanning Bessel beam was used here for densely populated fluorophores detected at a relatively low background level (Supplementary Fig. 5a).

**Fig. 2:**
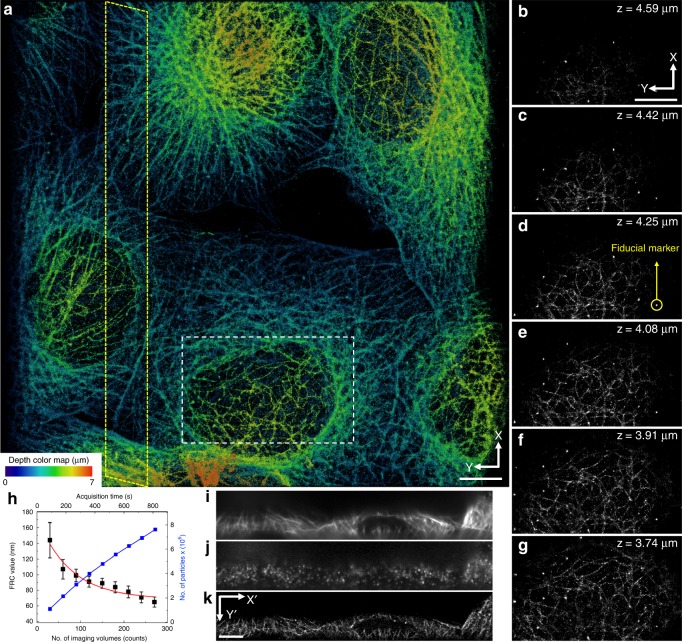
3D Large-scale localization imaging by Bessel lightsheet confinement. Super-resolution images of MC3T3-E1 cell lines with microtubule labeling (**a**) multiple cells plotted as maximum intensity projection over the entire imaging volume and (**b**–**g**) series of spatially sectioned sub-area enclosed by a white box illustrating the continuously optical sectioned images along the *z*-axis with an interval of 0.17 μm. **h** The Fourier ring correlation (FRC) was plotted with respect to the imaging volume used in the calculation of the image reconstruction. **i**–**k** A comparison of raw images within a sub-area (see **a**) enclosed in the tilt yellow box indicate the image orientation as shown in the yellow region in the inset of Fig. 1 are observed with regular lightsheet (**i**), localization-based lightsheet (**j**), and reconstructed image of the localization-based lightsheet (**k**). The raw image volume is composed of 192 × 672 pixels, 151 *z*-planes at 20 ms exposure time each, and a *z*-stack within a total of 3.02 s. The reconstructed image is made of 270 localization volumes in 15 min (all the scale bars: 5 µm)

Next, to demonstrate the 3D super-resolution imaging over a large imaging volume, microtubules (*β*-tubulin) from mouse MC3T3-E1 cells and hippocampal neurons from rat pup brain cultured on 5-mm coverslips were stained with primary (monoclonal anti-*β*-tubulin) and secondary (HMSiR labeled goat anti-mouse) antibodies. The dense labeling of *β*-tubulin with HMSiR blinking fluorophores enables the single-molecule localization for super-resolution imaging. As shown in Supplementary Fig. 6b, improved image resolution and image contrast were achieved by the designed localization-based imaging system as juxtaposed with the regular Bessel lightsheet microscope (see Supplementary Fig. 6a). Figure 2 shows super-resolution images of MC3T3-E1 cell lines over a large imaging scale at the speed of 1.8 × 10^4^ µm^3^ s^−1^ including multiple (see Fig. 2a) and continuously optical-sectioned cell images (see Fig. 2b–g). Figure 2a clearly shows large-scale super-resolution images revealing the visible intracellular fine structures of MC3T3-E1 cell lines via dense labeling of *β*-tubulin with HMSiR blinking fluorophores. A series of continuously optical-sectioned images of a sub-area (see Fig. 2a, enclosed in white box) along the *z*-axis with an interval of 0.17 µm were performed to reveal the good optical sectioning capability of the designed localization-based imaging system (see Fig. 2b–g). By tracing a fiducial marker (see Fig. 2d and Supplementary Fig. 6b, enclosed in yellow circle) adhered on the cell surface, the actual localization precision was determined at 40 and 45 nm in the *x*-axis and *y*-axis, respectively. The use of fiducial markers allowed us to improve the theoretical localization uncertainty to 20 nm laterally in the designed localization-based microscope system. The low fluorescence background and high signal-to-noise ratio were due to the optical confinement of scanning Bessel beam lightsheet revealing subcellular structures even in a complicated environment or overlapping regions of the cell. This efficient observation of the sub-diffraction distribution of protein molecules and physiological morphology of cell structures are of great value for studies of complex cellular interactions.

Fourier ring correlation (FRC) analysis was performed to estimate the lateral resolution^38,39^ (Fig. 2h) of the 3D reconstructed super-resolution images. The lateral resolution derived from the FRC analysis indicates feature with the highest spatial frequency that could be resolved in the reconstructed image; therefore, one should not confuse the structural resolution derived from FRC value with the optical resolution embedded physically in the imaging system. As expected from statistical certainty, the lateral resolution increased, respectively, by accumulating more frames for the reconstruction until reaching the barrier imposed by unavoidable background noises.

Our microscope had a FRC resolution of ∼107 nm after accumulating 75 consecutive volume frames and a resolution of ∼75 nm with 250 volume frames (see Fig. 2h). To retard resolution growth, 250 volume frames were accumulated for a 3D image with sufficient resolution without wasting valuable acquisition time. To demonstrate the resolution achieved by the designed localization microscope system, a sub-area (see Fig. 2a, enclosed in yellow box) was analyzed to compare the difference between the images acquired with regular lightsheet and localization microscope system (see Fig. 2i, j, respectively). A reconstructed super-resolution image (see Fig. 2k) was imaged by the designed microscope system.

**Fig. 3:**
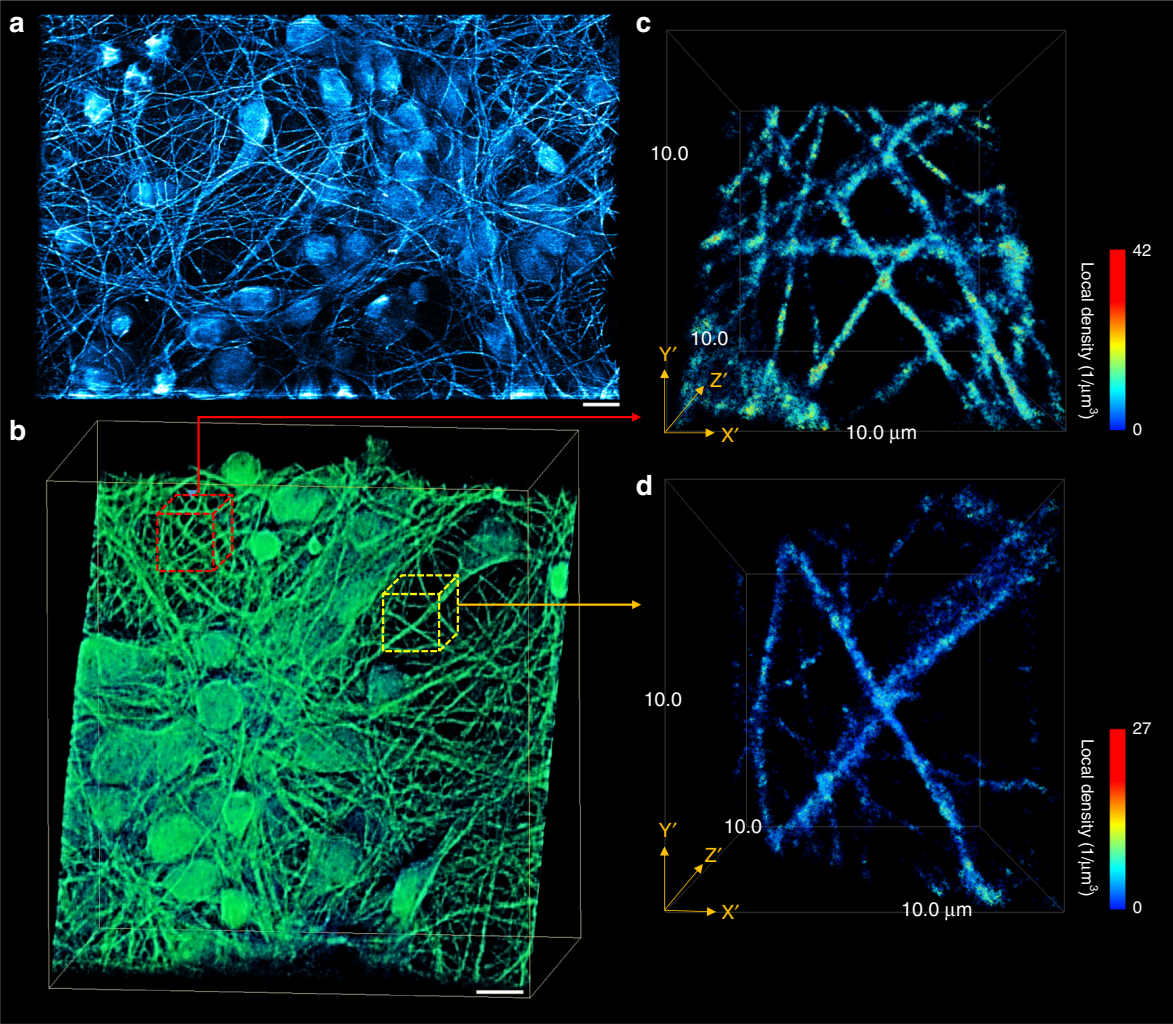
Astigmatism assisted to improve axial resolution for 3D lightsheet localization imaging. Representative images of the reconstructed three-dimensional super-resolution images of primary cultured neurons. **a** 3D maximum intensity projections of multiple neurons shown in a large field-of-view. Scale bar is 10 µm. **b** Reconstructed 3D super-resolution image of neurons; the two subareas (**c**, **d**) are enlarged to show a detailed structure of the intersected tubulin network. The pseudo-color scheme represents the density within a fluorescent 100-nm diameter sphere

Here, optical sectioning was determined by the thickness of the full width at half maximum (FWHM) of the central lobe of the Bessel beam corresponding to ~0.5 µm in the current configuration. The precise location of each fluorophore within the optical-sectioned layer could be identified via the introduction of optical astigmatism. Figure 3 shows the 3D super-resolution image of microtubules in primary cultured neurons from rat pup brain tissue. The microtubules in neurons were labeled with antibodies for localization-based imaging as described above. The incorporation of the HMSiR blinking fluorophore on *β*-tubulin reveals resolved neuronal structures including the cell body or soma, dendrites, axons, and axon terminals (see Fig. 3a). The sub-diffraction localization along the *z*-axis is resolved by fitting the distortion of the PSF with respect to *z*. Figure 3b shows that three-dimensional super-resolution imaging of neurons can be clearly resolved in the designed microscope system revealing the structure of tubulin filaments even within an interconnected and heavily overlapped network of neurons (see Fig. 3c, d). By extracting the line profiles of certain locations of tubulin filaments (shown in Supplementary Figs. 6 and 7), we found a fine structure with a dimension of *∼*100 nm is resolved in 3D by the microscope system.

### 3D live localization of the nuclear pore proteins

**Fig. 4:**
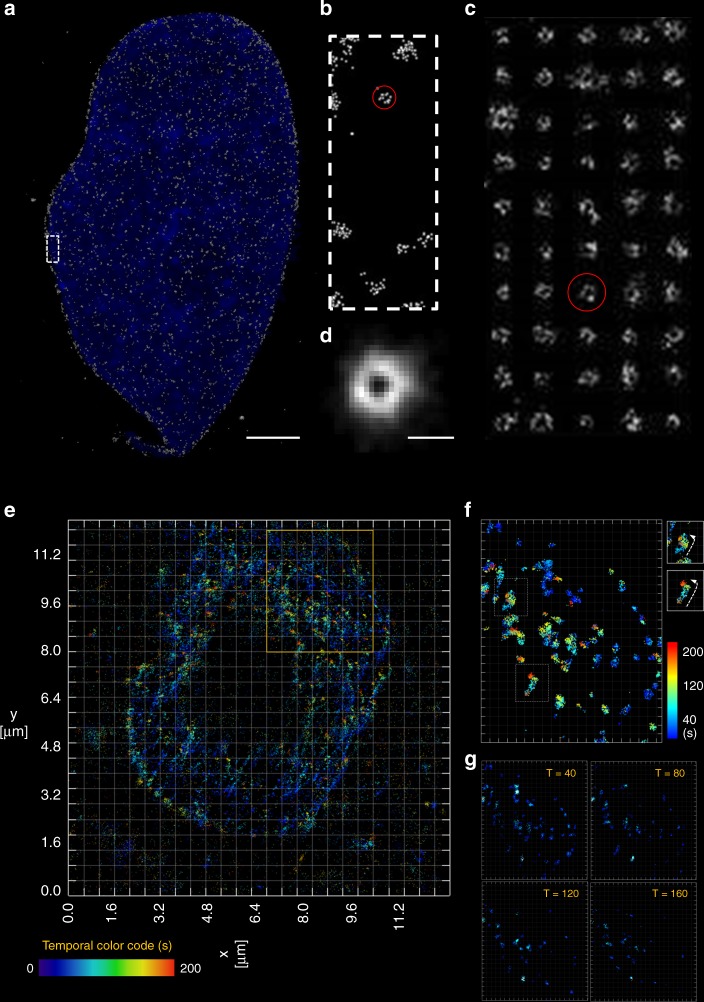
Fixed and live 2D localization of nucleoporins-POM121 labeled with blinking fluorophores. Fixed and time-lapse images of the reconstructed three-dimensional distribution of nucleoporin molecules. **a** 3D maximum intensity projections for NPCs structures of a fixed and intact HeLa cell expressing POM121-Halo and labeled with Halo-HMSiR dyes. The localized sites are displayed as a white point cloud while DAPI fluorescence nuclear staining is colored in blue. Scale bar is 3 µm. **b** The high magnification area of the white box in (**a**) with one NPC structure marked in red. After fitting by a donut function using least-square method, the top 50 optimal fits (shown as the montage in (**c**)) were centered and overlaid together as (**d**) with scale bar 100 nm. **e** The 2D reconstructed image on a live cell with POM121 labeled with HMSiR reconstructed based on 10,000 frames. The local density was plotted with temporal color code. **f** A magnified area in (**e**) is enlarged to show the temporal evolution of the distribution of NPC. Two selected cases are plotted with moving directions in the insets. **g** Images reconstructed from certain time interval are serially plotted to reveal change of the spatial distribution with respect to time (the contrast of (**f**, **g**) are adjusted to enhance the clustered feature of NPC)

To further demonstrate that our technique can obtain super-resolution images in 3D, we investigated the distribution of nuclear pore complex (NPC) proteins (POM121 or Nup153) on both fixed and living cells (Fig. 4). The labeling was done with HMSiR-Halo dye conjugated with HaloTag-labeled nucleoporins. The maximum intensity projection for the POM121 proteins on the fixed HeLa cell nucleus is shown in Fig. 4a. The sites of POM121 proteins are represented as the gray point cloud overlay; the DAPI-labeled nucleus is in blue. The enlarged view of the selected white area in Fig. 4a is depicted in Fig. 4b, where fine structures in the NPC clusters can be seen. To reconstruct these fine structures, we chose 100 NPC clusters on the nuclear membrane (50 of them are shown in Fig. 4c similar to the NPC cluster circled in red in Fig. 4b) for overlay in Fig. 4d. The reconstructed NPC structure is used to estimate the diameter at ~100 nm. The criteria for selecting NPC cluster candidates is described in the “Methods” section^40^. Although we could resolve the NPC pore diameter over an intact nucleus by lightsheet localization microscopy, the performance is expected to be compromised while attempting to image live image, such as NPC structure dynamics due to the sample movement or deformation. Since localization process is an accumulation of multiple images over time to resolve the nanostructure, the motion-induced artifacts will be expected for 3D live localization image where even higher speed is desired. We then selectively illuminated a single layer of nucleus by lightsheet at 20 ms exposure per frame for localization. Figure 4e shows a 2D mapping of the HMSiR-Halo dyes conjugated to Halotag-POM121 protein expressed on the nuclear membrane surface on NIH 3T3 cells. The image is rendered with temporal color code to visualize the time-dependent movement of POM121 protein. Figure 4f is a specified area in Fig. 4e, is enlarged to show the details of the clustered structure of POM121 protein. In Fig. 4g, the time-lapse image series of 10,000 frames (total length = 200 s, see Supplementary Movie 1) are segmented into a subset of 2000 frames (40 s). The images reconstructed from the subsets are plotted with respect to the time interval. The changed NPC location observed in each interval is likely related to (i) the global movement of the cell or (ii) the relative movement of NPC on the nuclear membrane surface. In certain case denoted in (Fig. 4f), we can track the transport of NPC by manual segmentation (as shown in the inset).

**Fig. 5:**
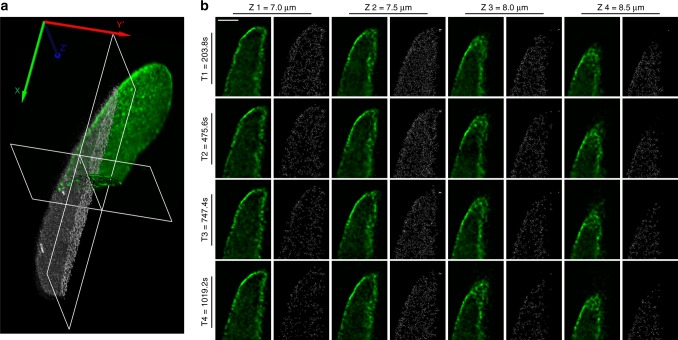
Live 3D localization of nucleoporins-Nup153 labeled with blinking fluorophores. **a** A 3D live localization of nucleoporin Nup153 expressed in HeLa cell nucleus was transfected with Halotag-EGFP-Nup153 plasmid (green overlay) and labeled with Halo-HMSiR dyes (gray overlay). Scale bar is 10 µm. **b** EGFP signal of Nup153 (green) is shown explicitly at four different Z slices (each were 0.5 μm apart, *Z*1 = 7 μm, *Z*2 = 7.5 μm, *Z*3 = 8 μm, and *Z*4 = 8.5 μm above coverslip) at different time points (*T*1 = 203.8 s, *T*2 = 475.6 s, *T*3 = 747.4 s, and *T*4 = 10,192.9 s). Localization results of HaloTag-labeled Nup153 proteins (gray) are accumulated from 40 time points each (*T*1 ± 133.6 s, *T*2 ± 133.6 s, *T*3 ± 133.6 s, and *T*4 = 10,192.9± 133.6 s). Scale bar is 3 µm

Our current system is capable of imaging the blinking molecules at up to 50 planes per second with the detectable signal-to-noise ratio, and it is insufficient to continuously follow the rapid morphological changes at nanoscale in 3D. A transition to 2D imaging of a single plane on the specimen is feasible to resolve the nanostructure. Instead of resolving well-defined structures such as aforementioned microtubules or NPC structure, lightsheet localization could have the advantage for mapping the distribution of the interested proteins in 3D^41,42^. In contrast to POM121 proteins that are presumably located at the nuclear periphery, Nup153 proteins reside not only on the nuclear envelope but also inside the live cell nucleus^43^. Therefore, to monitor the 3D live distribution of nucleoporin Nup153 inside the cell nucleus, HeLa cells were transfected with Halotag-EGFP-Nup153 and were visualized with the imaging system as shown in Fig. 5a. For comparison, a two-color acquisition was used in this experiment where the green fluorescent channel was used to monitor the EGFP signals for regular lightsheet microscopy; the red fluorescent channel from HMSiR-Halo dyes was used for localization-based microscopy. Figure 5b is the live mapping of Nup153, where 3D live images can be visualized through green florescence signals shown in green whereas the locations of individual Nup153 proteins at different time points were reconstructed from the HMSiR-Halo dyes signals shown in gray. Our results suggest that HMSiR-Halo fluorophore is suitable for localization microscopy with lightsheet illumination to image the entire cell nucleus in live cells for up to 43 min (see Supplementary Movie 2). These findings confirm that this is a gentle bioimaging tool for live localization because of the reduced phototoxicity.

## Discussion

An important issue in the single molecule super-resolution microscopy using lightsheet illumination is matching the lightsheet confinement and the depth of the field formed by separate excitation and detection objectives. To achieve better optical sectioning, a thinner lightsheet (<1 μm) is preferred and a higher numerical aperture (NA) collection objective (>1.1 NA) is required to obtain better resolution. To date, most single molecular studies that incorporate lightsheet illumination have focused on maximizing the collection efficiency of weak single-molecule signals using high numerical-aperture objectives (>1.2 numerical-aperture). However, the short working distance (~200 μm) and large physical profile of high numerical-aperture objectives prevent efficient coupling of the excitation and detection objectives in orthogonal objectives geometry^44^. To relax the steric constraint, the resulting lightsheet is brought to the focal plane via a high numerical-aperture objective by pseudo-TIRF illumination^45^, a fabricated reflection surface close to the sample position^46–48^, or beam steering by a prism^36,49^ at the cost of a thicker lightsheet (several micrometers), limited field of view, and 3D scanning stability. Here, 3D single-molecule localization over whole cells is achieved by minimizing the lightsheet thickness to improve the sectioning capability with acceptable collection efficiency using a 1.1 numerical-aperture detection objective in conventional SPIM setup^24,35^.

For SPIM, the excitation lightsheet can either be a real lightsheet created via a cylindrical lens or a virtual lightsheet generated by a scanning beam or dithering beam array^50^. A higher power density is required to detect the weak fluoresce signals from individual HMSiR dye molecules versus conventional fluorescence microscopy using fluorescent proteins or dye labeling. To create a lightsheet with high power density, the virtual lightsheet formed by a scanning beam is favored because it has enough peak intensity to pass the spontaneous blinking threshold of HMSiR dye molecules. Given that it has the same lightsheet length, a Gaussian lightsheet is usually much thicker than a Bessel lightsheet resulting in poor sectioning capability for the 3D localization microscopy. Lattice lightsheets on the other hand, utilize coherently interfered Bessel beams to remove side lobes of Bessel beams—thus, they can highlight subcellular dynamics in bulky cells while providing better signal-to-noise ratio.

However, for single-molecule localization microscopy, Bessel lightsheets are a better choice than lattice lightsheet due to better sectioning in Bessel excitation as discussed in the Supplementary Information (see Supplementary Fig. 1). For single-molecule detection with ultra-thin lightsheet confinement and high numerical-aperture objective collection, Supplementary Fig. 1c top view shows the narrower axial FWHM of the overall PSF for Bessel lightsheet illumination. This helps define the valid range for the presence of a target molecule. In addition, the lattice beam array is generated from spatial light modulator, and this diffraction process is less efficient (~3%) in terms of incident laser power loss (most of the power falls into unwanted diffraction orders). Based on these two arguments, we generated the Bessel beam by an annulus mask with ~10% power throughput (or even higher ones depending on the laser beam expansion ratio). We then conjugated the beam to the back focal plane of customized excitation objective through 4*f* configuration to eliminate the need for a spatial light modulator and hence simplify the design.

While the Bessel beam could create an ultrathin lightsheet below the diffraction limit for localization-based imaging, unwanted excitation by the Bessel beam side-lobes occurs due to the remaining intensity on the side-lobes. To overcome this challenge, an optical astigmatism was introduced by adding a cylindrical lens in our detection path to remove the fluorophores from the Bessel beam side-lobes that led to a large fitting uncertainty and thus were discarded in the image reconstruction. Therefore, the introduction of optical astigmatism could act as a filter for the rejection of the out-of-focus fluorescence background signals. Moreover, with the high density labeled spontaneously blinking HMSiR fluorophore, the overall resolution and the localization density increase rapidly at the first 200 frames (shown in Supplementary Fig. 8) so that fast localization process over 3D is feasible. Versus conventional photo-activation super-resolution techniques that employ shorter wavelength excitation, the HMSiR blinking dyes can be localized and imaged by longer wavelength (*λ* = 637 nm) to reduce the associated phototoxicity or possible alterations in the morphology of physiological cell structures in live localization applications.

In summary, we demonstrated the ability of the designed lightsheet localization microscope to acquire fine cellular structures over 3D within a relatively short imaging acquisition time. The main challenges in the acquisition of live cell images are minimizing the photodamage effect while retaining a good signal-to-noise ratio and providing a suitable environment for the cells or tissues to replicate their physiological structures^51^. Thus, the reported Bessel beam plane illumination microscope along with the HMSiR blinking dyes could be a promising approach for live 3D localization imaging.

## Methods

### Large-scale localization-based microscope system

The experimental setup of the assembled microscope system is shown in Fig. 1. The selective plane illumination microscope was equipped with three excitation lasers (*λ* = 488, 567, 637 nm) and two dichroic filters (DF1, DF2) that can be optimized for each fluorophore via wavelength selection with an acousto-optical tunable filter (AOTF). After passing through the AOTF, the laser beam is expanded to a diameter of 4 mm via the beam expanders (L7 and L8, L9, and L10) providing an equal Gaussian beam intensity distribution on the annular ring pattern of the custom-made annulus mask. The relay pairs of achromatic lenses (L11 and L12, L13, and L14) in a 4*f* arrangement were used for the conjugation of the annular ring pattern beam to a set of galvanometer scanners: GM-*X* and GM-*Z*′ (Cambridge Technology, 6215H) for the scanning of *x* and *z*′-directions, respectively. After passing through the galvanometer scanning mirrors, the ring-pattern laser beam is magnified by a pair of relay lens (L15 and L16) and projected to the rear focal plane of the excitation objective lens (Special optics, NA = 0.66, WD = 3.74 mm). As a result, a self-reconstructive Bessel beam was created via the optical interference mechanism. The created Bessel beam confines the laser energy within the illumination plane while conserving a sufficiently long propagation length^35^.

In the excitation arm setup, an appropriate NA of the excitation objective was used for the creation of a Bessel beam with a FOV length along the beam propagation that is comparable to the thickness of the specimen to be observed in the sample scan method; NA_out_ = 0.50 and NA_in_ = 0.42 were used for our experiments with an excitation laser at 637 nm^35^. Note that the back aperture was underfilled to have tradeoffs among the Bessel beam length, thickness, and side-lobe background. The way we varied the NA for creating different Bessel beam lengths at the back pupil of the excitation objective is by positioning the appropriate annulus on the mask (shown in Supplementary Fig. 2a) to filter the expanded Gaussian beam from laser source resulting in the desired annular ring pattern, which is conjugated to the back focal plane of excitation objective. For the MC3T3-E1 cell lines and neuronal observations, the length of the generated lightsheet was set to 20 μm to allow complete coverage of the cell intersection at an incident angle of 32.5° with respect to the sample coverslip. Hence, the thinness of the generated lightsheet allowed an effective background rejection and minimum levels of photobleaching.

The detection arm setup used a water-immersion objective (Nikon, CFI Apo LWD 25XW, NA = 1.1, WD = 2 mm) orthogonally oriented to the illumination plane and mounted on a piezo scanner (Physik Instrumente, P-726 PIFOC) for the collection of fluorescence signals. This was then imaged with a sCMOS camera (Hamamatsu, Orca Flash 4.0 v2 sCMOS) through a tube lens to have ~63× magnification resulting in a pixel size of 102 nm of the camera image plane. A weak astigmatism for sub-diffraction imaging in the axial direction was introduced by inserting a pair of cylindrical lens (Thorlabs, LK1002RM-A, LJ1516RM-A) between the tube lens and camera. The associated distortion of the PSF was then recorded by imaging fluorescent beads (ThermoFisher) with a z-step size of 40 nm and calibrated by the fitting with a defocusing model for the derivation of PSF shape dependence with respect to the axial direction. The power density in units of W cm^−2^ at the sample was estimated by assuming uniform illumination through a cross-sectional area encompassed by a X-scanning range of specified length^24^.

### Cell culture and growth

The MC3T3-E1 cell lines were obtained from the Institute of Biomedical Sciences, Academia Sinica. Hippocampal neurons were cultured via the papain-medium dissociation method^52,53^. Here, glial cells were used as conditional medium for neurons from the brain of post-natal rat pups (P0–P1, post-natal day 0–1). The glial cells were acquired from the brain cortex while the neurons were obtained from the hippocampus of the rat pup brain. The confluent glial cells were differentiated for the collection of secreted growth factors, i.e., the conditional medium required for the hippocampal neuron growth medium and maturation. Subsequently, the hippocampal neuron immunofluorescence staining was performed after the synaptogenesis on the 15th day of in vitro culture (15 *DIV*).

### Labeling microtubules with primary and secondary antibodies

The microtubules of the cultured cells were labeled with primary and secondary antibodies including the monoclonal anti-*β*-tubulin and HMSiR-labeled goat anti-mouse, respectively. First, the cells were fixed with a fixation solution containing 4% paraformaldehyde (Electron Microscopy Sciences, 15710), 4% sucrose (Sigma-Aldrich, S0389), and 1× phosphate buffered saline (PBS; Sigma-Aldrich, P3813). The cells were incubated at room temperature for 20 min and washed for three rounds with PBS. Second, the cells were permeabilized with permeabilization buffer containing a blocking solution and 0.1% of Triton X-100 (Sigma-Aldrich, T8787). They were incubated again for 15 min. Third, the cells were blocked with blocking solution containing 2% bovine serum albumin (Sigma-Aldrich, A1933), 4% normal goat serum (Thermo Fisher Scientific, 16210064), and 1× PBS, and then incubated again for 15 min. Finally, the cells were cultured on 5-mm diameter coverslips and labeled with primary and secondary antibodies: monoclonal anti-*β*-tubulin antibody from mouse (Sigma-Aldrich, T4026, dilution factor = 1000) and HMSiR-labeled goat anti-mouse whole IgG (Tebu-Bio, A202-01; dilution factor = 500; working concentration = 10 μg mL^−1^), and Alexa 488-labeled goat anti-mouse whole IgG (Thermo Fisher Scientific, A-11001; dilution factor = 5000; working concentration = 400 ng mL^−1^), then incubated overnight at 4 °C. After incubation, the cells were washed with PBS and stored in a 24-well plate with 1 mL per well of PBS until imaging experiments.

### Sample mounting and image acquisition

The cultured and labeled cells on 5-mm diameter coverslip (either MC3T3-E1 cell lines or hippocampal neurons) were mounted and fixed on the sample chamber of the designed large-scale localization-based imaging microscope system. For drift correction, the fiducial marker (0.1 μm red fluorescent beads) was introduced after cells were fixed, incubated with 2 μL of the fluorescent bead solution with 10,000× dilution from stock concentration for 10 min and later washed free of unbound beads. Epi-illumination mode was switched on to determine the precise location of the image of interest. In this configuration, the lightsheet excitation mode was switched-off, and epi-illumination was performed using the epi-objective. The sample was then scanned to determine the precise imaging location. When the location of the image of interest was determined, the configuration was changed to lightsheet excitation mode. A continuous lightsheet scan was performed via the excitation objective, and the optimum lightsheet position was defined for super-resolution imaging. To obtain a 3D super-resolution image of microtubules, the sample was scanned continuously through an automatic 3D axis sample stage, and the fluorescence signals were detected via a detection objective to providing a series of 2D z-stack images. Subsequently, the acquired 2D z-stack images were processed and analyzed for 3D super-resolution image reconstruction.

### Cell line expressed with Nup153 or POM121

The HaloTag-EGFPC3-Nup153 plasmid was generated by first amplifying the HaloTag sequence using pHTN HaloTag(R)CMV-neo (Promega, G772A) as the template; a paired primer (forward 5′-TTTGCTAGCAAAGCCACCATGGCAGAAA-3′ and reverse 5′-TTTACTAGTGCGTTATCGCTCTGAAAGTACAG-3′) were designed to add NheI and SpeI cleavage sites to facilitate subcloning. After double digestion by restriction endonucleases (NheI and SpeI), the product inserts into NheI-cleaved pEGFPC3-Nup153 (Addgene plasmid 64268) via the compatible-cohesive-ends. The live cell samples were Hela or NIH/3T3 cells that expressed HaloTag-EGFPC3-Nup153, using Lipofectamine 2000 (ThermoFisher, 11668027). The Hela or NIH/3T3 cell line that stably expressed Pom121-HaloTag was a gift from Dr. Yasushi Okada (RIKEN, Japan). Hela cells expressing HaloTag-EGFPC3-Nup153 or Pom121-HaloTag were seeded on a 5-mm diameter coverslip and cultured in a culture medium containing either 10 or 100 nM HMSiR-Halo for 16 h before the experiments.

### Image processing and analysis

Localization time-lapse images were analyzed by ThunderSTORM^54^, an ImageJ plugin with a customized macro for cluster processing. Prior to image analysis, the volumetric TIFF stacks were transposed into a time series sequence for each layer. System drifts will inevitably occur during the course of image acquisition, fiducial markers tracking or cross correlation was introduced to compensate, therefore, the entire analysis was preceded in the time domain to simplify the correction computations. To efficiently ingest these massive datasets, all images were transferred to a remote Lustre storage and subsequently analyzed by a four-node Torque cluster (Intel Xeon X5660 with 48 GB memory each connected to this Lustre storage).The results from each layer were then reduced into one single list on one of the cluster nodes. To present the density map of localization events, each particle is represented by a symmetric Gaussian function with a standard deviation equal to the theoretical localization uncertainty. For 3D volume rendering, the histogram of particle density map could be smoothed by a Gaussian kernel to reduce the required discontinuity.

### Select NPC candidates

The point cloud was binned into a pixelated image (Fig. 4b) using an effective pixel size of 10 nm. The candidate mask was first initiated by selecting pixels that had localized events. The holes were then filled morphologically in this preliminary mask. The mask was then dilated by a 3 × 3 square structure element to remove all pixel clusters that were not between 250 and 300 squared-pixels. Centroids were then determined for each pixel clusters and cropped a 32 × 32 region-of-interest from the aforementioned pixelated image for visualization (Fig. 4c).

### NPC averaging

The cropped region-of-interest used bivariate kernel density estimation^40^ to predict the most probable event probability distribution as Gaussian mixtures, without assuming data parametric model during the process, which eliminate the possibility of having specification bias. The actual center was then deduced by computing the centroid of the 2-D probability map; the cropped image was shifted accordingly and accumulated with each other (Fig. 4d).

### Reporting summary

Further information on experimental design is available in the Nature Research Reporting Summary linked to this article.

## Supplementary information

Reporting Summary

Description of Additional Supplementary Files

Supplementary Information

Supplementary Movie 1

Supplementary Movie 2

## Data Availability

The authors declare that the data supporting the findings of this study within the paper and its Supplementary Information Files are available from the corresponding author upon request.

## Code availability

The source code is available (https://bitbucket.org/account/user/cbc-group/projects/LOC).
