# Supplementary material for: Lightsheet localization microscopy enables fast, large-scale, and three-dimensional super-resolution imaging
Source: Commun Biol. 2019 May 9;2:177. doi: 10.1038/s42003-019-0403-9 (PMC6509110; doi:10.1038/s42003-019-0403-9)
Supplement: Supplementary file 1 — Reporting Summary [file 42003_2019_403_MOESM1_ESM.pdf]

nature research

Corresponding author(s): Dr. Bi-Chang Chen (Academia Sinica)

Last updated by author(s): Mar 20, 2019

Reporting Summary

Nature Research wishes to improve the reproducibility of the work that we publish. This form provides structure for consistency and transparency in reporting. For further information on Nature Research policies, see Authors & Referees and the Editorial Policy Checklist.

Statistics

For all statistical analyses, confirm that the following items are present in the figure legend, table legend, main text, or Methods section.

n/a	Confirmed	
☒	☐	The exact sample size (*n*) for each experimental group/condition, given as a discrete number and unit of measurement
☐	☒	A statement on whether measurements were taken from distinct samples or whether the same sample was measured repeatedly
☒	☐	The statistical test(s) used AND whether they are one- or two-sided<br>*Only common tests should be described solely by name; describe more complex techniques in the Methods section.*
☒	☐	A description of all covariates tested
☒	☐	A description of any assumptions or corrections, such as tests of normality and adjustment for multiple comparisons
☐	☒	A full description of the statistical parameters including central tendency (e.g. means) or other basic estimates (e.g. regression coefficient) AND variation (e.g. standard deviation) or associated estimates of uncertainty (e.g. confidence intervals)
☒	☐	For null hypothesis testing, the test statistic (e.g. *F*, *t*, *r*) with confidence intervals, effect sizes, degrees of freedom and *P* value noted<br>*Give P values as exact values whenever suitable.*
☒	☐	For Bayesian analysis, information on the choice of priors and Markov chain Monte Carlo settings
☒	☐	For hierarchical and complex designs, identification of the appropriate level for tests and full reporting of outcomes
☒	☐	Estimates of effect sizes (e.g. Cohen's *d*, Pearson's *r*), indicating how they were calculated

Our web collection on statistics for biologists contains articles on many of the points above.

Software and code

Policy information about availability of computer code

Data collection	All acquired data were carried out by SPIM, a LabVIEW-based system licensed from Janelia Research Campus, HHMI.
Data analysis	Localization was performed primarily by ThunderSTORM, accompanied by series of custom written script to execute on a self-hosted cluster. The ThunderSTORM version we used was development release 2016-09-10-b1 (https://github.com/zitmen/thunderstorm/releases/tag/dev-2016-09-10-b1) with FIJI. Custom written script was released on Bitbucket (https://bitbucket.org/account/user/cbc-group/projects/LOC).

For manuscripts utilizing custom algorithms or software that are central to the research but not yet described in published literature, software must be made available to editors/reviewers. We strongly encourage code deposition in a community repository (e.g. GitHub). See the Nature Research guidelines for submitting code & software for further information.

Data

Policy information about availability of data

All manuscripts must include a data availability statement. This statement should provide the following information, where applicable:

- Accession codes, unique identifiers, or web links for publicly available datasets
- A list of figures that have associated raw data
- A description of any restrictions on data availability

Due to TB range data size, it is hard to find a suitable long-term online repository, they are available upon request but requires further discussion on data transportation method.

Field-specific reporting

Please select the one below that is the best fit for your research. If you are not sure, read the appropriate sections before making your selection.

☒ Life sciences ☐ Behavioural & social sciences ☐ Ecological, evolutionary & environmental sciences

For a reference copy of the document with all sections, see nature.com/documents/nr-reporting-summary-flat.pdf

Life sciences study design

All studies must disclose on these points even when the disclosure is negative.

Sample size	The formation of each figure relies on the accumulation of images on the same position in the time series (about 200~400 time points). The formation of a 3D image is based on the z-axis limit formed by the length of the lightsheet and the size of the XY plane is limited by the size of the camera and the moving distance of the piezoelectric stage.
Data exclusions	Based on earlier works that used electron microscope and X-ray crystallography, we can use them to estimate a reasonable dimension range in unit super-resolved pixel. We can also further subtract noises based on camera product specification. Details are described under "Select NPC candidates" in the methodology section.
Replication	All fluorescence signals in experiments were depleted upon termination, therefore, for each sample, we are not able to repeat the acquisition. But following the sample preparation steps, we were able to consistently achieve the stated parameters in the text, such as resolution limitation respect to acquisition duration.
Randomization	Randomization was not required in our experiments, since no statistical hypothesis was imposed.
Blinding	No grouping was performed in our experiments, hence, blinding was not applicable in our design.

Reporting for specific materials, systems and methods

We require information from authors about some types of materials, experimental systems and methods used in many studies. Here, indicate whether each material, system or method listed is relevant to your study. If you are not sure if a list item applies to your research, read the appropriate section before selecting a response.

Materials & experimental systems

n/a	Involved in the study	
☒	☐	Antibodies
☒	☐	Eukaryotic cell lines
☒	☐	Palaeontology
☒	☐	Animals and other organisms
☒	☐	Human research participants
☒	☐	Clinical data

Methods

n/a	Involved in the study	
☒	☐	ChIP-seq
☒	☐	Flow cytometry
☒	☐	MRI-based neuroimaging
