# Supplementary material for: Lightsheet localization microscopy enables fast, large-scale, and three-dimensional super-resolution imaging
Source: Commun Biol. 2019 May 9;2:177. doi: 10.1038/s42003-019-0403-9 (PMC6509110; doi:10.1038/s42003-019-0403-9)
Supplement: Supplementary file 2 — Description of Additional Supplementary Files [file 42003_2019_403_MOESM2_ESM.pdf]

Description of Additional Supplementary Files

File Name: Supplementary Movie 1
Description: 2D live localization of cell expressed with Halotag-POM121

Two-dimensional live cell localization animation shows a stable cell line expressing Halotag-POM121 (a subunit of nuclear pore complex) treated with Halo-HMSiR. The animation records the position and dynamics of the intracellular nuclear pores. The left panel is the localization results of the Halotag-POM121 displayed with 20 successive images and each image is reconstructed based on 500 frames with an exposure time 20 ms. The right panel shows the accumulation for the 20 time-lapse images to demonstrate the dynamics of the NPC. Grid size: 1μm, total duration is about 200 seconds. Color shows the localization density.

File Name: Supplementary Movie 2
Description: 3D live localization of cell expressed with Halotag-EGFP-Nup-153

Three-dimensional live localization microscopy animation shows a cell transfected with Halotag-EGFP-Nup153 (a subunit of nuclear pore complex) treated with Halo-HMSiR. The animation shows the two-color time-lapse data EGFP and HMSiR signals for Nup153 protein excited by wavelength 488nm and 637 nm, respectively. The upper part of the left panel shows the EGFP signals of Nup153 and the lower part shows the accumulative processed localization image for every 40 frames and the right one shows the localization results from HMSiR signals reconstructed from every 40 frames in depth color code. The total duration of imaging process is approximately 40 minutes for 350 volumes with two color recorded.
