# Supplementary material for: Lightsheet localization microscopy enables fast, large-scale, and three-dimensional super-resolution imaging
Source: Commun Biol. 2019 May 9;2:177. doi: 10.1038/s42003-019-0403-9 (PMC6509110; doi:10.1038/s42003-019-0403-9)
Supplement: Supplementary file 3 — Supplementary Information [file 42003_2019_403_MOESM3_ESM.pdf]

**Supplementary Figures**

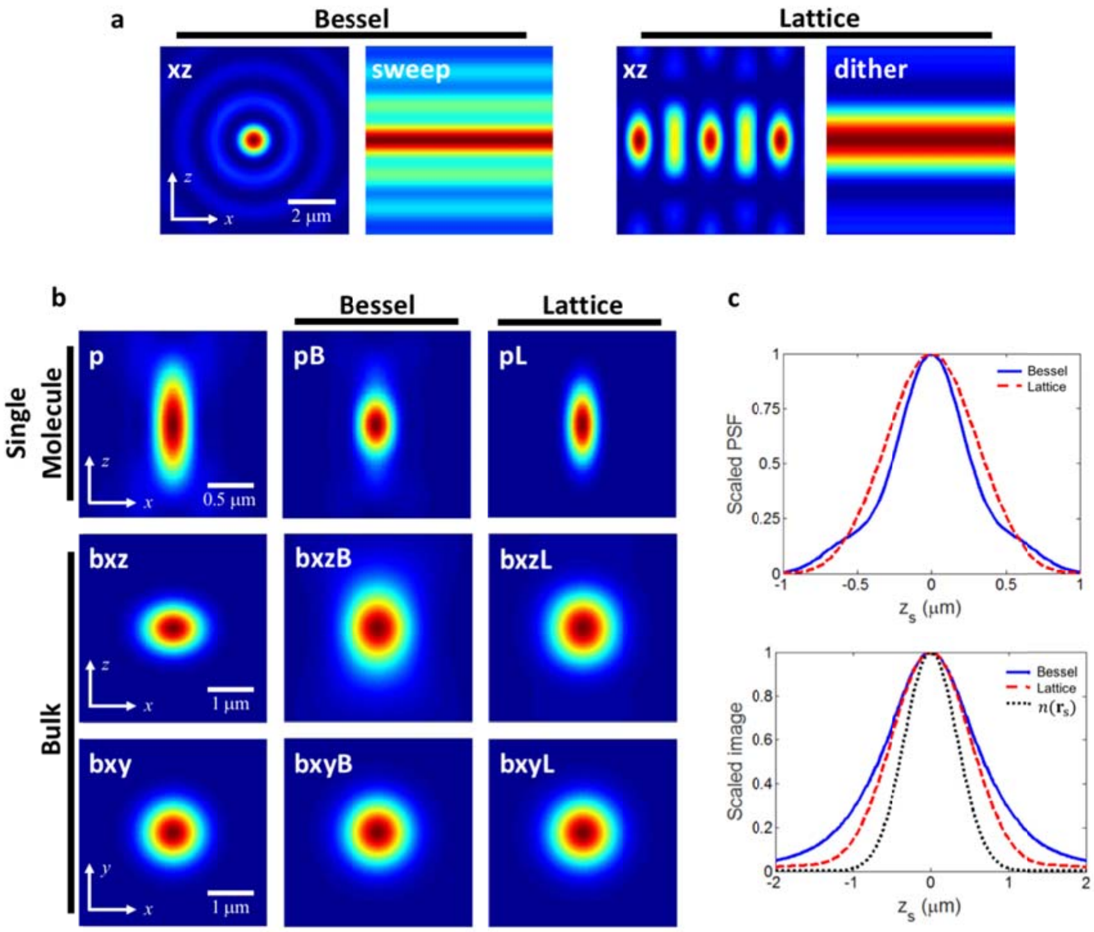


**Supplementary Figure 1. (a) The cross sectional intensity profiles of Bessel beam and lattice light on the $xz$ plane. The two patterns extend sufficiently long along the $\pm y$ directions. The (sweep and dither) panels are respectively the excitation patterns corresponding to the scanning of Bessel beam and lattice light. The intensity profile of Bessel lightsheet has more significant side-lobes in the axial direction. (b) The cross sectional intensity profiles of (p) detection PSF, (pB) overall PSF of Bessel lightsheet, and (pL) overall PSF of lattice lightsheet on the $xz$ plane. The three PSFs are all symmetric around the axial direction. The two overall PSFs are both sharper than the detection PSF. While the axial FWHM of the overall PSF of Bessel lightsheet is smaller than that of lattice lightsheet, the related tails and side-lobes outside the main peak are also more significant. (bxz, bxy) The cross sectional profiles of fluorophore density along $xz$ and $xy$ plane. (bxzB, bxyB) and (bxzL, bxyL) are images retrieved with Bessel lightsheet and lattice lightsheet on different planes respectively. While the lateral broadening brought by Bessel and lattice lightsheets are similar (bxyB and bxyL), the axial broadening induced by lattice lightsheet is less serious (bxzB and bxzL). (c, top) The scaled overall PSFs of Bessel (solid blue) and lattice lightsheets (dashed red) as a function sample position $z_s$ ($x_s = y_s = 0$). The axial**

**FWHMs of Bessel and lattice lightsheets are 0.54 and 0.72 μm, respectively. (c, bottom) The scaled images as retrieved with the Bessel (solid blue) and lattice lightsheet (dashed red) along the axial direction though the focal point ($x_s = y_s = 0$). The scaled fluorophore density distribution (dotted black) is also shown for comparison. The axial FWHM of the images associated with Bessel and lattice lightsheets are 1.36 and 1.19 μm, respectively.**

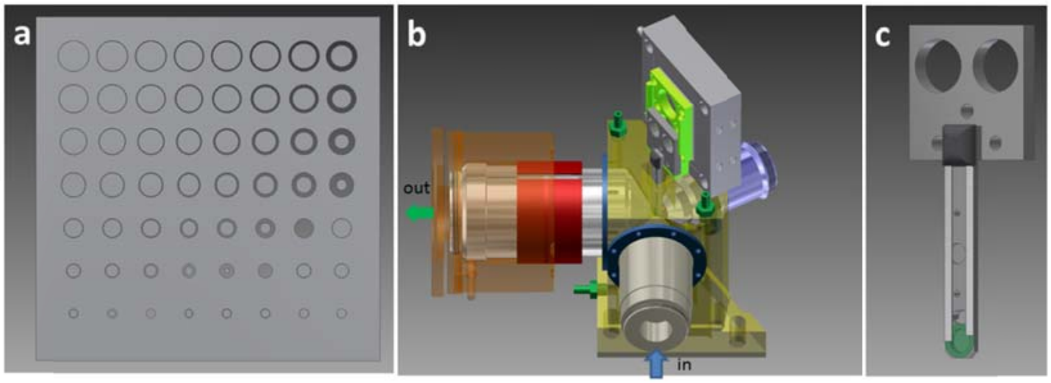


**Supplementary Figure 2. (a) Apodization mask contains an array of annuli array of different outer and inner diameters for generating Bessel beams of different lengths and thicknesses (b) schematic of the specimen chamber, which consists of the chamber itself, excitation objective (in), detection objective (out), the specimen holder, and the wide-field view-finding objective. (c) The zoom in view of the sample holder showing the 5 mm coverslip in green is held by two elastic steel stripes.**

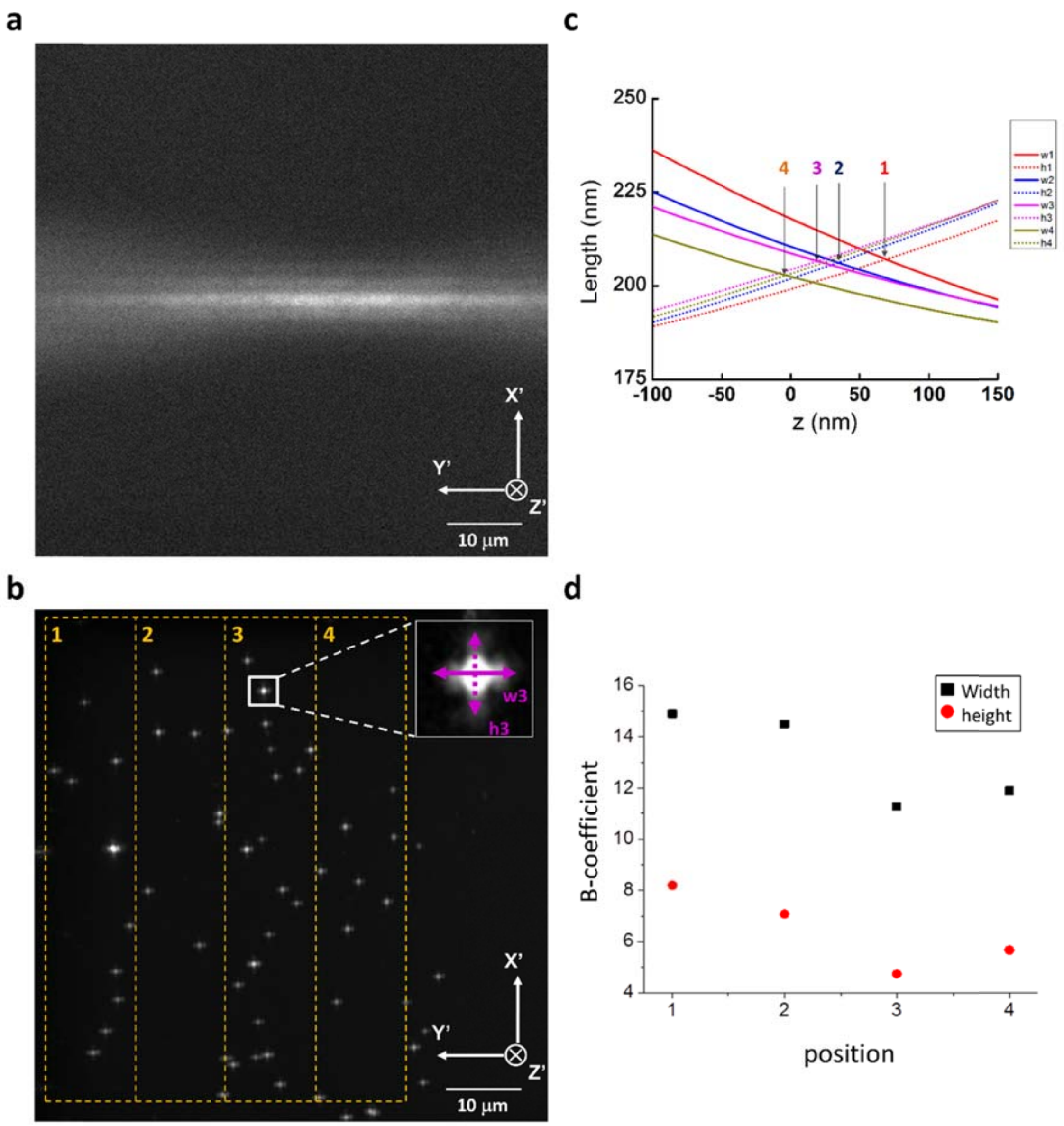


**Supplementary Figure 3. Calibration curves at different regions of lighsheet: (a) Bessel beam profile used for the experiments (b) maximum intensity projection of multiple beads imaged by scanning Bessel beam shown in (a) by detection objective-scanning. (c) The Z calibration curves across different regions of the Bessel beam profile. (d) The fitting quality related B-coefficient for different positions.**

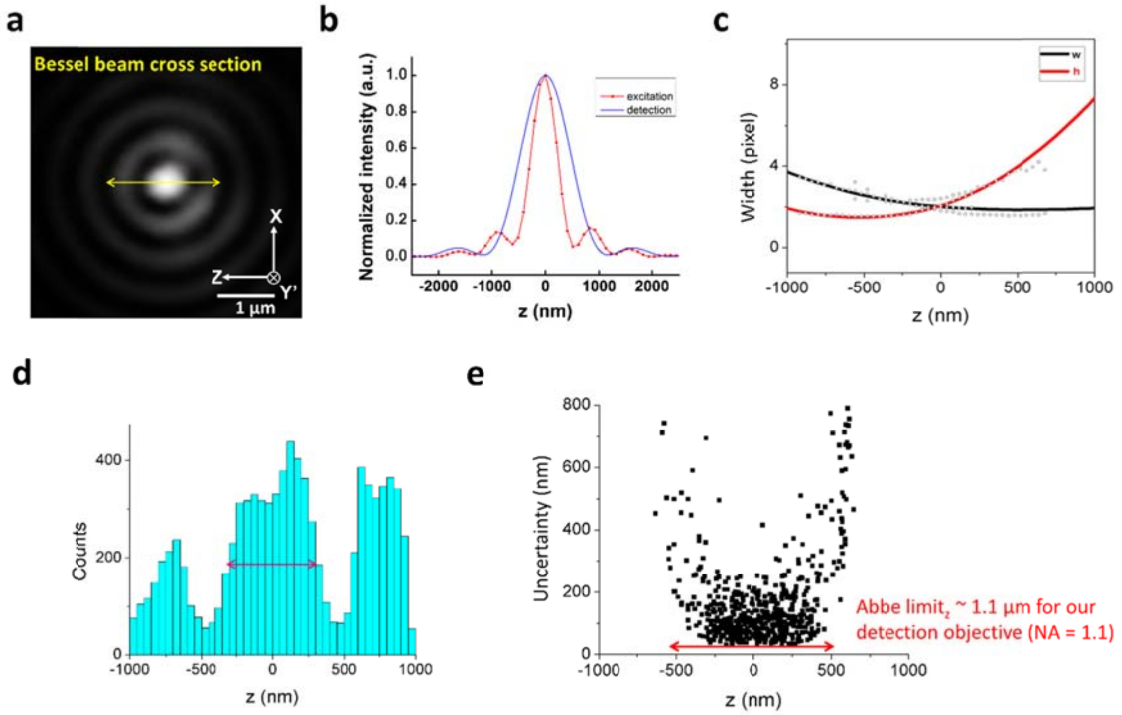


**Supplementary Figure 4. (a) The excitation profile (xz PSF) of the Bessel beam. (b) The line profile along the yellow line in Supplementary Figure 4(a) is shown in red; theoretic detection PSF for 1.1 NA objective is shown in blue (c) The calibration curve used in localization calculation (d) The population of the localized particles along the *z*-direction (e) The axial dependency of the localization in *z*.**

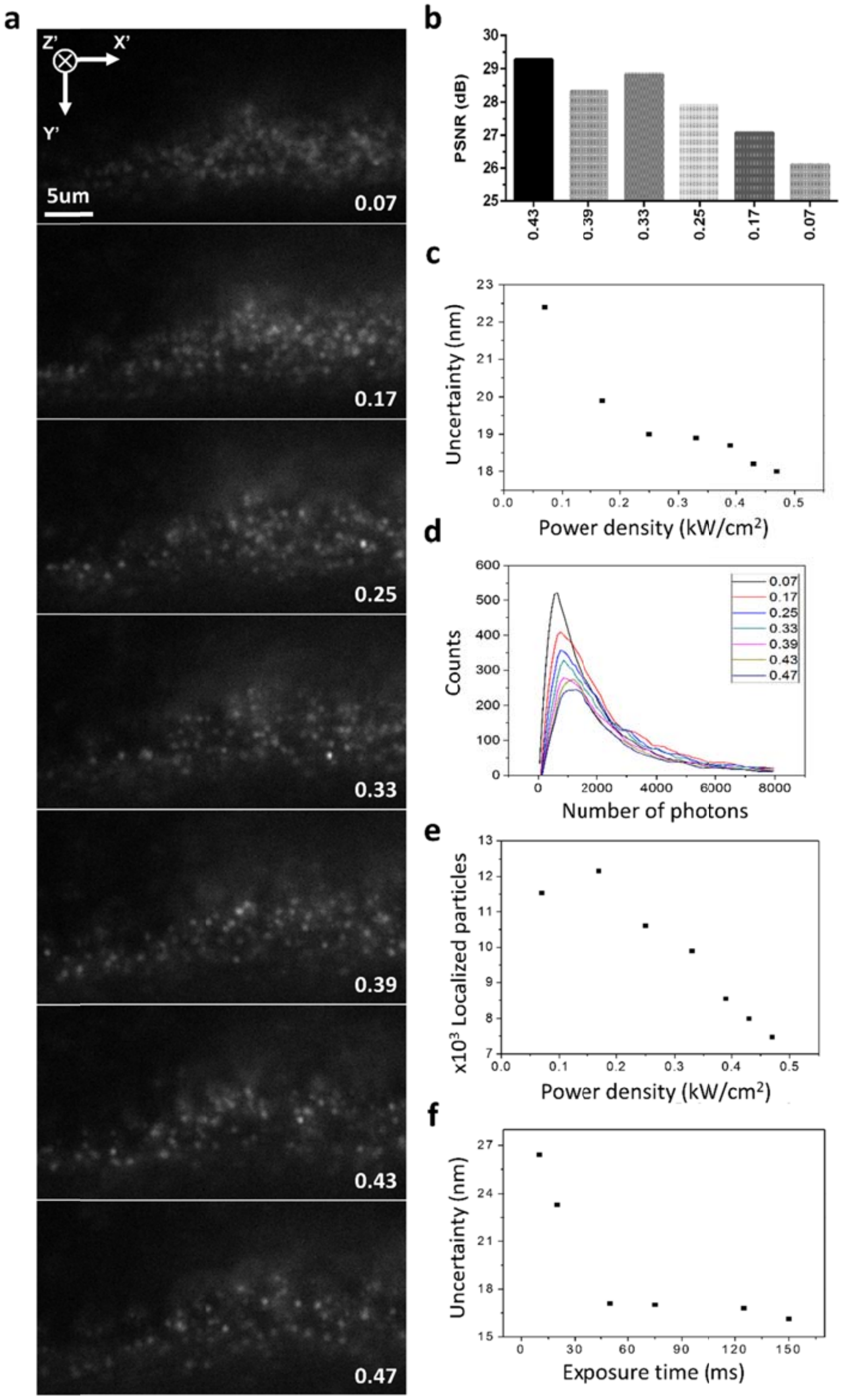


**Supplementary Figure 5. (a) raw images of a single slice of cell labelled with microtubule-HMSIR at different powers (0.07, 0.17, 0.25, 0.33, 0.39, 0.43 and 0.47 (kW cm^-2) at 50 ms exposure time (b) peak signal-to-noise ratio (PSNR) expresses the ratio between maximum possible intensity and the power of distorted noise that potentially affects image**

**quality. Consider that all the images were blinking events under similar optical configurations aside from excitation power, PSNR was used as a metric to estimate significantly excited signals with respect to background fluctuations, which composes of Poisson noise from the camera and most importantly, background from side lobes.(c) localization uncertainty of the HMSiR blinking fluorophores dependence on power-density acquired within 50 millisecond exposure time (d) histogram of the photons collected from a single blinking event acquired at different power-density in a single volumetric scan of MC3T3-E1 cell (e) total number of localized particle at different power density from a single volumetric scan (f) localization uncertainty of the HMSiR blinking fluorophores dependence on the exposure time at the power density of 0.47 kW cm^-2.**

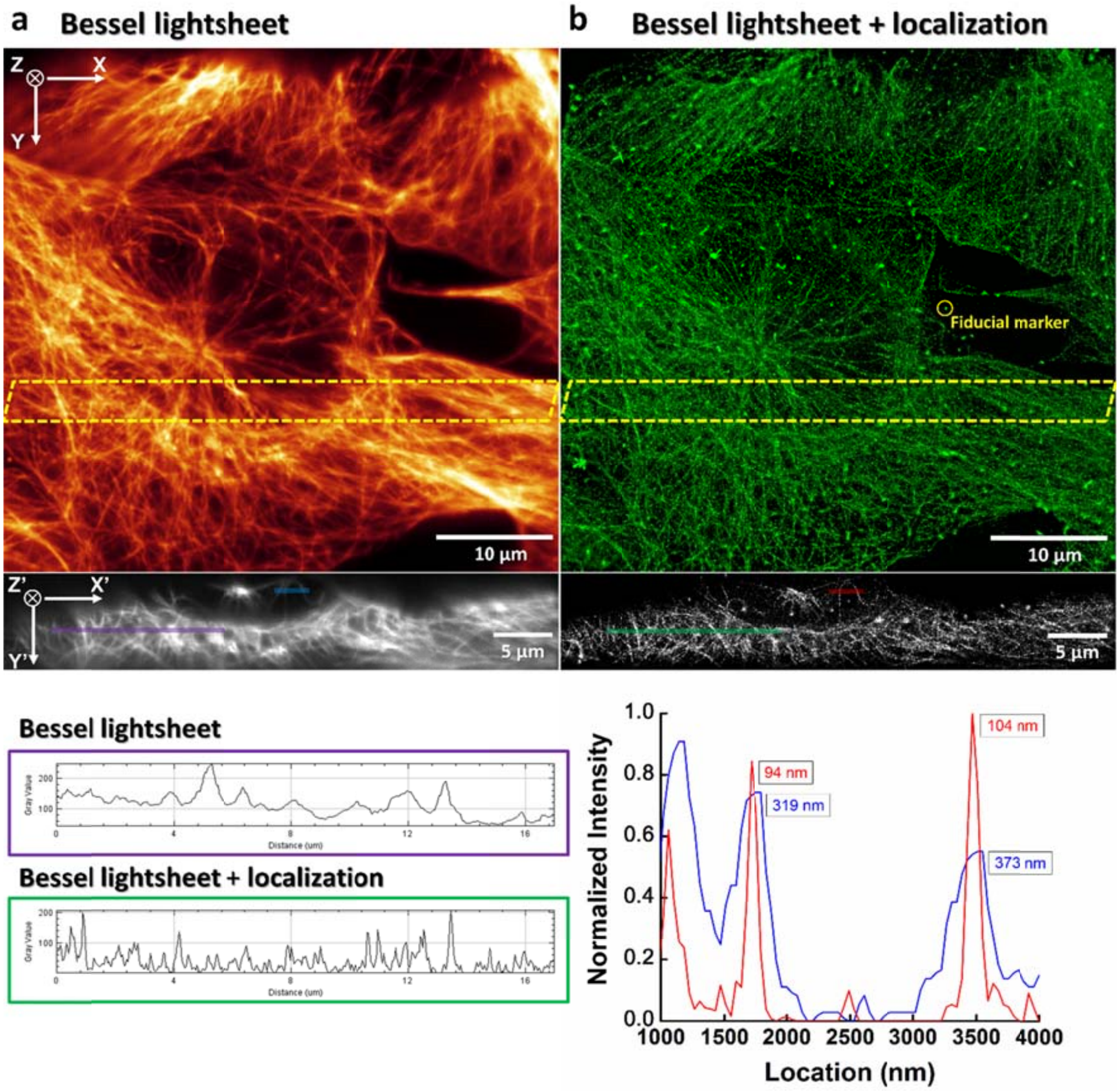


**Supplementary Figure 6. A comparison was made between the microtubule images observed with (a) Bessel light-sheet fluorescent microscope (λexc = 488 nm) and (b) localization-based microscope system (λexc = 637 nm). The fiducial marker is enclosed in the yellow circle. The scale bar is 10 µm. The image volume is composed of 192 x 512 pixels, 121 z-planes at 20 ms exposure time for each z-plane within acquisition time of 2.42 s. The reconstructed image is accumulated with 600 3D localization volumes. The line profiles of the same area (yellow box) taken by two methods shown in purple and green boxes, separately. Inset shows the zoom in area for a selected structure with linecut profile demonstrating the resolution improvement from ~300 nm to ~90 nm).**

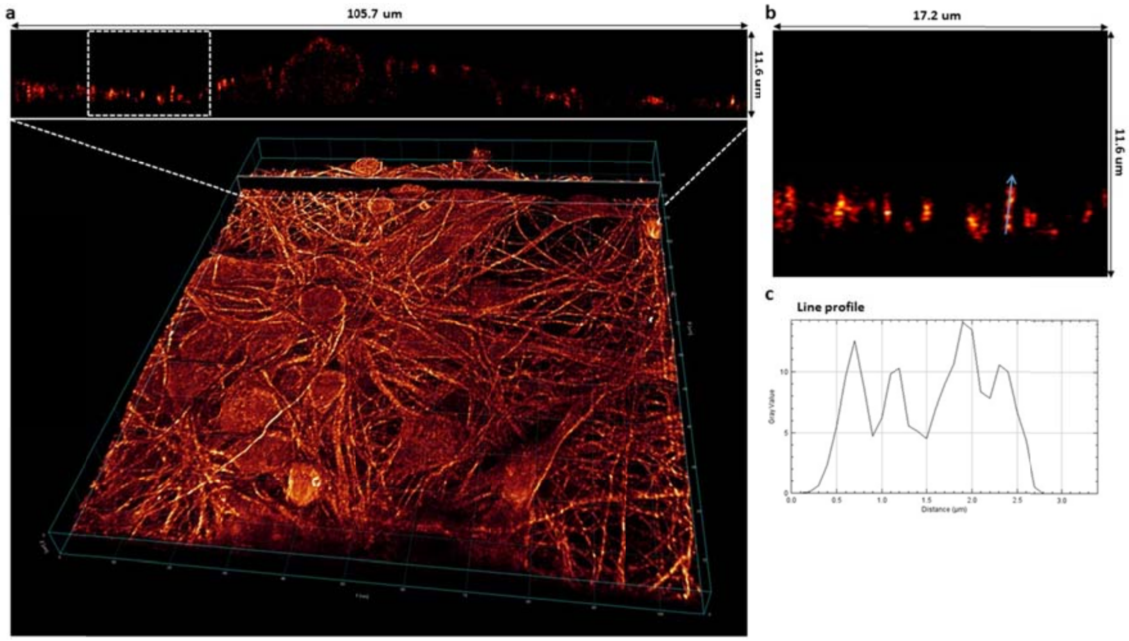


**Supplementary Figure 7. The XZ plane line profile of 3D super-resolution image of microtubules in primary cultured neurons from rat pup brain tissue (a) A orthoslice of the 3D data presented in Fig. 3(b), with an area of ~100 x10 μm^2 (b) the zoom-in view of the selected white box shown in (a) with fine structures in axial direction (c) the line profile along with the blue line in (b) with the FWHM ~ 100 nm that cut through series of microtubules.**

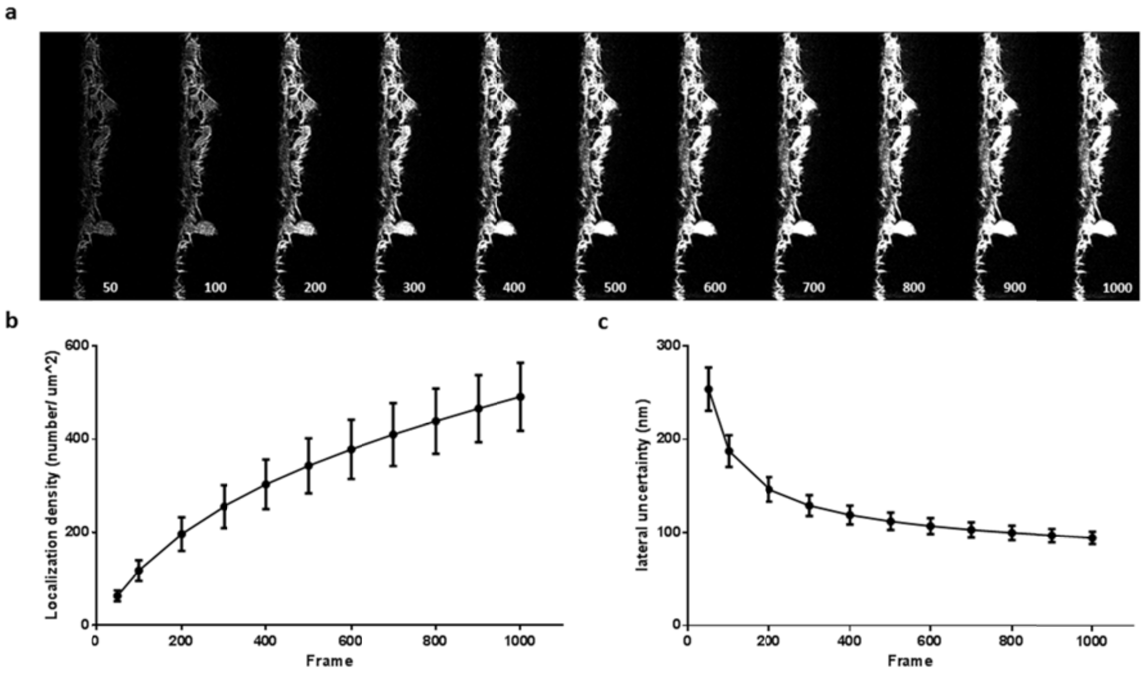


**Supplementary Figure 8. The analysis of the lateral uncertainty with respect to the localization density. (a) The reconstructed images of neuron tubulin fibers using different number of frames. (b) The localization density and (c) the lateral uncertainty are plotted with respect to the frames used in image reconstruction.**

## Supplementary Note

### Superiority of Bessel light sheet for single-molecule detection over lattice lightsheet

The sectioning capability and axial resolution of a lightsheet in selective plane illumination microscopy (SPIM) mainly depend on the thickness of its central peak and amount of power in the side-lobes. In general, the thinner central peak and weaker side-lobes of the lightsheet improve the performance of SPIM. These principles are common to the fluorescence detection in both the macroscopic and single-molecule regimes. However, the same lightsheet may work distinctly for different purposes of detections. A lightsheet suitable for imaging macroscopic fluorophore distributions might not be the best candidate for positioning of a single molecule. Here, we theoretically show that the lightsheet formed by sweeping a single Bessel beam (Bessel lightsheet) has the better chance to accurately localize the position of a single fluorophore. On the other hand, the lightsheet created through the dithering of lattice light (lattice lightsheet) is more useful for monitoring the profile of a macroscopic fluorophore distribution.

Supplementary Figure 1(a) shows the cross-sectional intensity distributions (on $xz$ plane) of a single Bessel beam and lattice light, respectively. The two excitation patterns are assumed to extend sufficiently long along the $\pm y$ directions (propagation direction of beams), and fluorescence detections are carried out at $+z$ or $-z$ side. The numerical generation of these two patterns was described in Ref. [1]. Here, the excitation wavelength is set to 0.637 μm, and the inner and outer numerical apertures (NAs) for the creation of these beam patterns are 0.42 and 0.5, respectively. For our experiment, the two types of excitations were scanned along the $x$ direction to form nearly homogeneous lightsheets on the $xy$ plane. In views of it, the two intensity profiles in Supplementary Figure 1(a) (sweep and dither) panels are averaged along the $x$ direction. The resulted patterns (sweep and dither) are intensity profiles of the lightsheets corresponding to a single Bessel beam and lattice light, respectively. The side-lobes along the axial ($z$) direction in Supplementary Figure 1(a) Bessel beam (sweep) panel are associated with the ring pattern of Bessel beam $xz$ profile in Supplementary Figure 1(a). A comparison between groups in Supplementary Figure 1(a) indicates that the side-lobes are less prominent on the excitation pattern of lattice light sheet.

In the confocal-microscopy-like (CM-like) operation of SPIM, the sectioning capability and axial resolution of a lightsheet are determined by an overall point spread function (PSF), which is closely related to the detection PSF and excitation pattern of lightsheet (also called excitation PSF). Given a sample position $\mathbf{r}_s$, the importance of overall PSF can be understood from the conjugate image $F(\mathbf{r}, \mathbf{r}_s)$ near the focal plane ($xy$ plane) at the object side:

$$F(\mathbf{r}, \mathbf{r}_s) = \int d\mathbf{r}' \, \mathrm{PSF}_{\mathrm{det}}(\mathbf{r} - \mathbf{r}')[I(\mathbf{r}')n(\mathbf{r}' - \mathbf{r}_s)], \quad \text{(Eq. 1)}$$

where $\mathrm{PSF}_{\mathrm{det}}(\mathbf{r}')$ is the detection PSF; $I(\mathbf{r}')$ is the excitation pattern of lightsheets; $n(\mathbf{r}' - \mathbf{r}_s)$ is the density profile of fluorophores referenced to sample position $\mathbf{r}_s$. For simplicity, we assume that the focal plane coincides with plane of maximal intensity of lightsheets, and the focal point is located at

the origin ($\mathbf{r}=\mathbf{0}$). In the CM-like mode, rather than recording the blurred profile of $F(\mathbf{r},\mathbf{r}_\mathrm{s})$ at each location $\mathbf{r}$, we keep track of $F(\mathbf{0},\mathbf{r}_\mathrm{s})$ as a function of the sample position $\mathbf{r}_\mathrm{s}$, which is virtually taken at the focal point $\mathbf{r}=\mathbf{0}$. In this way, equation (Eq. 1) is rewritten as

$$F(\mathbf{0},\mathbf{r}_\mathrm{s}) = \int \mathrm{d}\mathbf{r}'\mathrm{PSF}_\mathrm{det}(-\mathbf{r}')I(\mathbf{r}')n(\mathbf{r}'-\mathbf{r}_\mathrm{s})\Big|_{\mathrm{Let}\ \mathbf{R}=\mathbf{r}_\mathrm{s}-\mathbf{r}'}$$
$$= \int \mathrm{d}\mathbf{R}\ \mathrm{PSF}_\mathrm{all}(\mathbf{r}_\mathrm{S}-\mathbf{R})n(-\mathbf{R}) \qquad \text{(Eq. 2)}$$

where the overall PSF is defined as $\mathrm{PSF}_\mathrm{all}(\mathbf{R}) \equiv \mathrm{PSF}_\mathrm{det}(-\mathbf{R})I(\mathbf{R})$. In terms of sample position $\mathbf{r}_\mathrm{s}$, the signal $F(\mathbf{0},\mathbf{r}_\mathrm{s})$ is the convolution between $\mathrm{PSF}_\mathrm{all}(\mathbf{r}_\mathrm{s})$ and inverted density profile $n(-\mathbf{r}_\mathrm{s})$. During experimental operations, one could effectively change the sample position $\mathbf{r}_\mathrm{s}$ by moving the sample holder or optical setup of microscope. The former option was adopted in our experiment.

The detection PSF is modeled as the intensity distribution of a lens around its focal point as immersed in water. It is calculated based on the integral formulation in [2, 3] and fast Fourier transformation. The NA of lens is 1.1, and emitter wavelength is set to 0.660 μm for HMSiR molecules. The cross sectional profile of the detection PSF (on $xz$ plane) is shown in Supplementary Figure 1(b), panel (p). The detection PSF is symmetric around the axial ($z$) direction. The overall PSFs corresponding to Bessel and lattice lightsheets are shown in panel (pB) and (pL) of Supplementary Figure 1(b), respectively. Since the lightsheets are laterally homogeneous, the two overall PSFs are also axially symmetric. The lateral full-width-at-half- maxima (FWHM) of these three PSFs are close, indicating similar lateral resolutions. On the other hand, comparing the two overall PSFs to their detection counterpart, we see that the excitation pattern effectively reduces the thickness of overall PSFs along the axial direction, marking the better sectioning capability and axial resolution than those of the detection lens alone. In addition, the overall PSF of Bessel lightsheet has the smaller axial FWHM than that of the lattice lightsheet. However, the more prominent tails and side-lobes outside the main peak are also present on the overall PSF of Bessel lightsheet. These features distinguish the applicability of two lightsheets in the macroscopic and single-molecule detections.

For single-molecule detections, the important parameter is the axial FWHM of the overall PSF, which helps define the valid range for the presence of target molecule. Suppose that a single fluorescent molecule is located at $\mathbf{r}_\mathrm{s}$, namely,

$$n(\mathbf{r}'-\mathbf{r}_\mathrm{s}) = \delta(\mathbf{r}'-\mathbf{r}_\mathrm{s}). \qquad \text{(Eq. 3)}$$

In this case, the recorded pattern $F(\mathbf{0},\mathbf{r}_\mathrm{s})$ is simply the $\mathrm{PSF}_\mathrm{all}(\mathbf{r}_\mathrm{S})$. The smaller axial FWHM of $\mathrm{PSF}_\mathrm{all}(\mathbf{r}_\mathrm{S})$ then helps locate the position of molecule more precisely. In Supplementary Figure 1(c), top view, we show the scaled overall PSF $[\mathrm{PSF}_\mathrm{all}(\mathbf{r}_\mathrm{s})/\mathrm{PSF}_\mathrm{all}(\mathbf{0})]$ as function of $z_\mathrm{s}$ as the sample is moved vertically through the focal point ($x_\mathrm{s}=y_\mathrm{s}=0$). The axial FWHMs corresponding to the single-molecule detections based on Bessel and lattice lightsheets are 0.541 and 0.720 μm,

respectively, indicating that the Bessel light-sheet can provide the more precise fixation of molecule position along the axial direction.

On the other hand, for detections of a macroscopic distribution of molecules, the long tails and relatively significant side-lobes outside the main peak of the overall PSF corresponding to the Bessel light-sheet are unwanted. As an example, let us consider a Gaussian density profile of fluorophores as follows:

$$n(\mathbf{r}' - \mathbf{r}_\mathrm{s}) \sim \exp\left\{-4\ln(2)\left[\frac{(x\prime - x_\mathrm{s})^2 + (y\prime - y_\mathrm{s})^2}{l_\mathrm{t}^2} + \frac{(z\prime - z_\mathrm{s})^2}{l_z^2}\right]\right\}, \quad \text{(Eq. 4)}$$

where $l_\mathrm{t} = 1$ μm and $l_z = 0.8$ μm are the lateral and axial FWHMs of the density distribution, respectively. The cross sectional profiles of Gaussian distribution on the $xz$ plane $(y' - y_\mathrm{s} = 0)$ and $xy$ plane $(z' - z_\mathrm{s} = 0)$ are shown in Supplementary Figure 1(b) panel (bxz) and (bxy), respectively. The corresponding cross sectional images as retrieved with sample scanning using the Bessel light-sheet are shown in Supplementary Figure 1(b) panel (bxzB) and (bxyB), respectively, and the counterparts based on the lattice lightsheet are illustrated in Supplementary Figure 1(b) panel (bxzL) and (bxyL). As shown from Supplementary Figure 1(b) panel (bxzB), (bxyB), (bxzL), and (bxyL), the images are blurred both laterally and axially in views of the original density profiles in Supplementary Figure 1(b) panel (bxz) and (bxy). While the images corresponding to both light-sheets exhibit similar lateral broadening, the axial broadening brought by Bessel lightsheet is more serious than that induced by lattice light-sheet. The significant tails and side-lobes on the profile of $\mathrm{PSF}_\mathrm{all}(\mathbf{r}_\mathrm{s})$ corresponding to Bessel light-sheet are the cause to axial blurring since they tend to mix the information from different vertical layers together. In Supplementary Figure 1(c), bottom view, we compare the scaled density profile $n(\mathbf{r}_s)$ and images corresponding to Bessel and lattice light-sheets along axial direction through the focal point ($x_\mathrm{s} = y_\mathrm{s} = 0$). The axial FWHMs of the images associated with the Bessel and lattice light-sheets are 1.363 and 1.199 μm, respectively, corresponding to 70.4% and 49.9% increments from the original FWHM of 0.8 μm. The axial resolution of the lattice light-sheet indeed outperforms that of the Bessel light-sheet under such circumstances. Therefore, for detections of macroscopic fluorophore distributions, the lattice lightsheet would provide the better axial resolution than Bessel lightsheet does.

In summary, depending on the mode of detections, different lightsheets should be adopted in imaging. The narrow axial FWHM of overall PSF corresponding to the Bessel lightsheet makes it a more powerful tool for single-molecule detection. On the other hand, the lattice lightsheet would be more useful for imaging macroscopic distributions of fluorophores due to the lower weight of its overall PSF outside the main peak.

**Detailed components of Bessel lightsheet localization microscopy setup**

The schematic of the optical system is shown in Figure 1. The beam from a laser combiner equipped with 488 nm (Coherent OBIS 488 nm LS 150 mW), 561 nm (Coherent OBIS 561 nm LS 150 mW),

and 637 nm (Coherent OBIS 637 nm LX 140 mW) lasers is expanded to a diameter of 4 mm by two lenses (8 mm FL/12.24 mm dia (L1, L3, and L5), Thorlabs C240TME-A, 20 mm FL/12.5 mm dia Edmund 47-661(L2, L4, and L6)) and combine each lasers with a mirror (Thorlabs, BB1-E02-10 - Ø1" Broadband Dielectric Mirror, 400 - 750 nm) and two dielectric filter (Semrock, Dichroic Filter (DF): DF1= LM01-503-25; DF2= Di03-R561-t1-25x36). The expanded laser beam uses an acousto-optic tunable filter (AA Quanta Tech, Optoelectronic AOTF AOTFnC-400.650-TN) to control the exposure time and the wavelength selection. The laser beam passed through the AOTF is expanded to a diameter of 6 mm using beam expander (Thorlabs, AC254-060-A (L7) and AC254-125-A (L8) Ø1" Achromat, 400 - 750 nm) to ensure an even Gaussian intensity distribution over the annular ring pattern on the customized aluminum coating (thickness of 1,500 angstroms) 406 synthetic quartz mask by lithography as shown in Supplementary Figure 2(a). A relay lens pair (Thorlabs, AC254-100-A (L11) and AC254-075-A (L12) Ø1" Achromat, 400 - 750 nm) was used to conjugate the ring pattern created on the mask to a set of galvanometer scanner (Cambridge Technology, 6215H) which is composed of a pair of achromatic lenses (Thorlabs, AC254-085-A (L13 andL14) Ø1" Achromat, 400 - 750 nm) aligned in a 4f arrangement. After passing through the scanning mirror set, the ring pattern is magnified through relay lens (Thorlabs, AC254-254-A (L15) and AC254-400-A (L16) Ø1" Achromat, 400 - 750 nm) and conjugated to the back focal plane of the excitation objective (Special Optics, 0.66 NA, 3.74 mm WD). The ring pattern is projected to the rear focal plane of excitation objective and form a self-reconstructive Bessel beam by optical interference. The energy of the beam is confined within the illumination plane while a sufficiently long propagation length is maintained. By positioning the appropriate annuls on the mask, the effective NA was chosen to create a Bessel beam with the beam FWHM length comparable to the thickness of the specimen. In this study, the length of the light sheet is set around 20 um for a complete coverage of the cell intersection at an incident angle of 32.5 degrees to the coverslip as shown in Supplementary Figure 2(b). Supplementary Figure 2(c) shows the zoom in view of our home-made sample holder to mount 5 mm round coverslip. The two thin elastic steel strips were glued by epoxy so that coverslip could be loaded and unloaded easily. The illumination created by scanning the Bessel beam could create a thin lightsheet, which is beneficial for background rejection and photo-bleaching. In Supplementary Figure 2(b), orthogonal to the illumination plane, a water immersed objective lens (Nikon, CFI Apo LWD 25XW, 1.1 NA, 2 mm WD) mounted on a piezo scanner (Physik Instrumente, P-726 PIFOC) is used to collect the fluorescence signal, which is then imaged through an emission filter (Semrock Filter: LP02-638RU and FF01-446/523/600/677) onto a sCMOS camera (Hamamatsu, Orca Flash 4.0 v2 sCOMS) by a 500 mm tube lens (Edmund 49-290, 500 mm FL/50 mm dia; Tube Lens). A weak

astigmatism used for sub-diffraction imaging in axial direction was introduced by inserting a pair of cylindrical lens (Thorlabs, LK1002RM-A, LJ1516RM-A) between the tube lens and camera.

**Z Calibration curves with astigmatism introduced in the setup**

It is very important to choose a suitable length of lightsheet to perform the 3D imaging especially for localization due to the balance of optical sectioning and signal-to-noise ratio. The criteria is to have a lightsheet length just enough to cover the size of the specimens. In Supplementary Figure 3(a) shows the lightsheet profile used to perform our lightsheet localization. As shown in the profile, Bessel beam has better optical confinement around the beam center; on the other hand, the regions away from the center have worse sectioning capability and higher background. Experimentally, we scanned this Bessel beam in X' directions to form a scanning lightsheet. To determine the Z calibration curve with astigmatism introduced in the detection path, we further scanned this lightsheet in Z' direction with synchronized detection objective mounded on piezo-stage. Shown in Supplementary Figure 3(b) is a maximum intensity (MIP) projection of all the planes collected along Z' direction. The Z calibrations curves for different regions are plotted in Supplementary Figure 3(c). The fitting quality related B-coefficients are depicted in Supplementary Figure 3(d). The purpose of these figures is to illustrate the quality of z calibration curve across different regions of the Bessel beam profile. As indicated in these figures, only region 3 and 4, which are closer to center of the Bessel beam, produce the best fitting results in objective scan configuration. To ensure all images are reconstructed from the best optically-sectioned regions, sample scan was used for all the images shown in the main text. In order to measure the astigmatism calibration curve, we imaged beads with a Z' step size of 40nm by objective scan. Supplementary Figure 3(b) is the MIP of a volume (pixel dimension 512x512x600, 52.7x52.7x24 um^3, in X'Y'Z' order) along detection objective axis. With the Bessel beam profile shown in Supplementary Figure 3(a), we divide the MIP image of beads into four regions: 1,2,3 ,and 4, where beads in regions 3 and 4 falls in the optimal region of the Bessel beam, with smaller B-coefficient. B-coefficient is the independent parameter used in the parabolic fitting in ThunderSTORM calibration curve analysis. It is simply the constant term in the parabola equation used to fit the defocusing of PSF [4, Supplementary Material]. The inset of Supplementary Figure 3(b) shows w3 and h3 represent width in Y' and X' axes in the region 3. Curves in Supplementary Figure 3(c) are fitted results from data obtained in Supplementary Figure 3(b) at different Z' positions.

**Bessel beam localization with astigmatism present**

Bessel beam has an issue about the side-lobe background. In order to understand how this affects our

localization process, Supplementary Figure 4(a) shows the XZ excitation profile of our Bessel beam, central lobe with concentric rings background. The way we characterized the Bessel beam cross-sectional intensity is to position a focus fluorescent bead around the center of the Bessel beam and use the x(z) galvos to raster-scan the excitation beam in 100-nm steps across the bead over 5-μm range in the *xz* plane while recording an image at each position. The cross-section intensity of the Bessel beam is calculated by the integrated fluorescence counts recorded from the bead in each image gives a measure of the intensity of the beam at the associated *xz* position. The image created by the entire *xz* set of these measurements yields the cross-section of the Bessel beam. The image should closely approximate an ideal Bessel beam, with symmetric side lobes as shown in Supplementary Figure 4(a). The yellow line profile along the Bessel beam is shown in Supplementary Figure 4(b) marked in red. Since for lightsheet configuration, the detection arm is separated from excitation arm, we also plotted the theoretical detection PSF for our collection lens (NA= 1.1 at the emission wavelength of 670 nm, see Eq. 5). As one can see the lightsheet confinement is better than the depth of focus of used detection objective, which gives us overall better sectioning capability resulting in the reduction of background contribution from the Bessel side-lobe. With the Z calibration curve obtained according to aforementioned procedure plotted in Supplementary Figure 4(c), the way we got the curve is to choose a potential peak based on the local maximum; afterward, width and height are determined by fitting an elliptical Gaussian model by maximum likelihood method in a radius of 4 pixels around that peak, initial sigma is set to 1.6 pixels. If the peak intensity is not unique, they will be registered as candidate dots (with close proximity between each other) for fitting (therefore, same depth, multiple points, but it must be multiple of 2), due to slightly difference in pixels, the fitting result will be slightly different as well. That's the reason why there are points well fitted and some are not. Note that the software we used for the calculation is ThunderSTORM. Please refer to the development release 2016-09-10-b1 (https://github.com/zitmen/thunderstorm/releases/tag/dev-2016-09-10-b1) with Fiji (rolling updated).

$$Abbe\ Limit = \frac{\lambda}{NA^2} \quad \text{(Eq. 5)}$$

Supplementary Figure 4(d) shows the population of the localized HMSiR molecules along the Z direction by use of the calibration curve shown in Supplementary Figure 4(c). As one can see, there are the contributions from main-lobe and side-lobe of Bessel beams, separately. Moreover, if we further consider the localization uncertainty of the HMSiR blinking fluorophores dependence on the axial position, shown in Supplementary Figure 4(e). The uncertainty becomes larger when the molecules sit outside from 0.8 um in axial direction (the help from excitation confinement and PSF of detection objective, see Supplementary Figure 4(b)), where the events will be discarded due to high

uncertainty so that we could keep the localization events with high precision in axial direction with the introduction of weak astigmatism. Another method reported by Fahrbach *et al* to suppress Bessel beams side-lobe background is the confocal slit readout [5]. Given that confocal slit detection is used in our imaging system (high NA for both excitation and detection path and 1 camera pixel = ~102 nm), the synchronization between the position of ultra-thin lightsheet (~half a micron) and camera imaging pixels (several pixels in width) will be very difficult. Since we are aiming for high-speed and large-scale localization imaging, this critical requirement prevents us from using slitted Bessel lightsheet for single-molecule localization imaging applications.

**Experimental parameters for Bessel beam localization with HMSiR molecules**

To evaluate the balance between the localization precision and image acquisition speed, a comparison has been made on the relationship of the total number of localized particles and the fitting uncertainty under different imaging parameters used in the volumetric scan of the HMSiR labeled microtubules. The raw images of a single slice of a cell labeled with HMSiR-microtubules imaged at different illuminated power densities (0.07, 0.17, 0.25, 0.33, 0.39, 0.43 and 0.47 kWcm^-2) and same exposure time are shown in Supplementary Figure 5(a). All the images have the same scale in intensity to show how the background from the Bessel side-lobe affects the signal-to-noise (SNR) ratio of single-molecule. In order to quantify this parameter, we use peak signal-to-noise ratio (PSNR) expressing the ratio between maximum possible intensity and the power of distorted noise that potentially affects image quality. Consider that all the images were blinking events under similar optical configurations aside from excitation powers, PSNR was used as a metric to estimate significantly excited signals with respect to background fluctuations, which composes of Poisson noise from the camera and most importantly, background from side lobes. The PSNR of different illuminated powers is shown in Supplementary Figure 5(b), using 0.47 kWcm^-2 case as the baseline. As plots show that the PSNR behaves similarly around the high power regime. The same for the localization uncertainty dependence on the power density is shown in Supplementary Figure 5(c). Due to better SNR, the higher precision of the localization can be obtained in the range of 0.17 to 0.47 kW cm^-2. The uncertainty of the Gaussian fitting for the single-molecule localization decreases with decaying excitation power density and consequently lowering the photons collected from a single localization event as shown in Supplementary Figure 5(d). On the other hand, it was observed that the total number of localized particles reaches a maximum value at a relatively low power density and dramatically drops with increasing laser power in Supplementary Figure 5(e). This result indicates that, even though higher excitation power could lead to more precise determination of particle

location for single-molecule localization, it would yield to a significant loss on the number of particles. Therefore, a moderate power density of approximately 0.17 to 0.33 kW cm^-2 was employed, wherein it is considered the optimum parameter value for the designed microscope system. A similar relationship was observed between the exposure time and the localization uncertainty, in which a window of exposure time of approximately 50 to 70 milliseconds was determined for an efficient image acquisition. When an exposure time outside the window was used, it would result in a decrease in localization uncertainty and longer image acquisition process without a noticeable improvement in localization precision and density (see Supplementary Figure 5(f). Moreover, in practical imaging applications, a longer imaging acquisition time will result in the downgraded resolution due to either imaging system drift or sample deformations. Therefore, we have to optimize all these parameters to have a fast 3D localization process with better precision. Compared to previously reported data acquired from the total internal reflection fluorescence (TIRF) microscope, the developed scanning Bessel beam light sheet is significantly capable of imaging the entire cell body rather than the adjacent plane in the close vicinity of the coverslip. In conclusion, for a high-speed localization-based imaging process necessary for live cell imaging applications, a localization precision with a fluctuation below ~27 nm can be achieved with an imaging speed of up to 2 sec volume^-1 (assuming 200 stacks in a single imaging volume). Moreover, the lifetime of the open form of the particles should be reduced while maintaining the brightness, to precisely determine the localization of fluorophores for high speed imaging.

### Comparison of the images from Bessel lightsheet and Bessel lightsheet localization

In order to make the comparison for the spatial resolution improvement by the localization process, Supplementary Figure 6 shows the cell imaging with microtubule labelled with Alex488 and HMSiR molecules. 3D cell image was acquired by Bessel lightsheet sample scan mode with the excitation of 488 nm laser as shown in Supplementary Figure 6(a). For Bessel lightsheet localization imaging, excitation wavelength of 637 nm is used to make HMSiR molecules blink so that the image shown in Supplementary Figure 6(b) can be reconstructed based on the localization. The exposure time for each plane (z-slice) composed of 192x512 pixels (1 pixel = 0.103 nm) was 20 ms, and each plane was 0.5 μm apart during the sample scanning. The raw volume was 192x512x121 voxels, which took 2.42 second to finish a volume acquisition. The entire volume was repeatedly scanned for 600 times in 24 minutes. The data of the same z slice for regular Bessel lightsheet and lightsheet localization indicated by yellow box is shown in the middle of Supplementary Figure 6. The line profiles along the purple and green line are shown in the corresponding boxes, showing the resolution improvement for the

same area. The detailed resolution comparison of two methods based on the line cut of the same structure along the blue and red lines in Supplementary Figure 6(a) and (b) separately, is shown in the bottom of Supplementary Figure 6. As one can see, the resolution improvement is from ~ 300 nm to 100 nm. Moreover, in order to see the axial resolution for fine structures in the designed system, Supplementary Figure 7 is a slice of our neuron dataset shown in Fig. 3(b), which shows an axial direction line profile with the FWHM ~ 100 nm that cut through series of microtubules.

### The analysis of the resolution with respect to the localization density

As shown in the Supplementary Figure 8, the overall resolution and the localization density increase rapidly at the first 200 frames. The speed of the accumulation of the density reduces thereafter because of the photo-bleaching and the depletion of the fluorophores, which suppresses the further increase of the overall resolution. In our data reported here, the lateral resolution reaches 94.6 nm ± 6.7 nm when 1000 frames (corresponding to 200 minutes) are used in acquisition. On the other side, the resolution soon increases to 150 nm within 200 frames, which enables the observation of certain fine structures of the neuron tubulin (shown in Supplementary Figure 8 (a)). The localization density and the overall resolution are plotted with respect to the frames used in image reconstruction as shown in Supplementary Figure 8 (b) and (c), respectively.

### Processing the pipeline

A quick and easy program was designed for the image processing and analysis of three-dimensional super-resolution image reconstruction of MC3T3-E1 cells and neurons. This section provides a detailed information of the series of sequential events on 3D image processing and analysis. The customized program was created to efficiently run the program pipeline using the available resources at hand.

1. **Input**

   **1.1** For the efficient processing of massive dataset, the 3D TIFF image stacks were transferred to a remote Lustre [6] storage and a subsequently analysis of 2D processing per slice was performed using a four-node Torque cluster (Intel Xeon X5660 with 48 GB memory each, connected to the Lustre storage) [7].

2. **Transpose**

**2.1** To perform drift correction in a single pass, the XYZ image stacks were converted to time series XYT image stacks on the acquisition workstation post-experiment since the ThunderSTORM [6] requires the users to provide the time series 3D TIFF image stacks.

**2.2** A straightforward C program would parse the file by libtiff [8] and re-write them back in round-robin fashion. The files in-process are not buffered in the memory first but directly concatenated to the new XYT stack for performance reason.

### 3. Accounting

**3.1** Accounting was performed on the head node of the Torque cluster. On this step, no data transfer was carried out since all the data are located on the local NAS storage.

**3.2** After the acknowledgment of the file list, they were evenly distributed to multiple parts which is determined by the number of available worker nodes. Partial file lists were assigned to each worker node for data transfer.

### 4. Transfer

**4.1** The partial file list was fetched to the rsync [9] utility and pulled from the local NAS to a temporary shared workspace (between worker nodes) created under home directory of the worker node, since /tmp space is not enough to store each batch of data.

**4.2** Though the user could hide the transfer latency through dynamic job submission, it would increase the complexity and could hardly keep the script robust enough for general user.

**4.3** Transfer and processing of data will not be terminated but rather ignored if some errors occur for certain files. Instead, the output will be recorded in a log file for later inspection and manual re-submission.

**4.4** Additional wrapper scripts, IJ macro and calibration file (required for 3D localization) were copied from the local NAS to worker nodes as well, in order to provide a minimalistic way to update scripts on the cluster. The introduction of additional transfer time is negligible.

### 5. IJ Macro

**5.1** While IJ [10] was still buggy in headless mode, therefore, XVFB (X virtual framebuffer) [11], an in-memory display server, was created by each worker node to contain each IJ instance.

**5.2** IJ reads the user preference file during initialization and creates one when it does not exist. This will cause race condition and put IJ in an unpredictable state therefore the user preference file is redirected to each workspace using JVM command line arguments.

**5.3** Temporary directory under /tmp was created for the CSV results to avoid excessive small batch data transfer. It was removed upon termination of the JVM instance by shell script trap.

**5.4** The macro detects the existence of calibration file, and called the respective 2D/3D configuration for ThunderSTORM and execute them for all the files in the workspace. The CSV files were dumped in per file basis, hence, each Z slice will have a respective output.

6. **Transfer**

**6.1** The CSV files were rsync back to the shared workspace for merging.

7. **Merge**

**7.1** The first worker, determined randomly by head node during resource request, would execute a Python script to merge the CSV files.

**7.2** A SQLite [12] database was created on the fly to hold all the CSV files of interest.

**7.3** For the 2D localization, the sequential *z* information was deduced by *z* step size and source file, since each *z* slice is contained in a single 3D stack (see "Transpose").

**7.4** NaN filtering and geometry manipulation, required by data acquired through sample scan, were also performed here.

**7.5** A new CSV file was generated by the final content in the database.

8. **Transfer**

**8.1**Prior to the transfer, the discrete CSV files and the database were removed.

**8.2** The resulting CSV was rsync back to the local NAS storage, under the source directory for further processing.

9. **Post-process**

**9.1**Additional processing happened here, *i.e.* ASH plot and FRC evaluation [13].

**Note:** Detailed information on image processing and analysis is provided in the source code [14].

## References


1. B. C. Chen et al., “Lattice light-sheet microscopy: Imaging molecules to embryos at high spatiotemporal resolution,” Science 346(6208), 1257998 (2014).
2. E. Wolfs, “Electromagnetic diffraction in optical systems - I. An integral representation of the image field,” Proc. Royal Soc. Lond. A, 253(1274), 349-357, (1959).
3. B. Richard and E. Wolfs, “Electromagnetic diffraction in optical systems, II. Structure of the image field in an aplanatic system,” Proc. Royal Soc. Lond. A, 253(1274), 358-379, (1959).
4. Oxford Academic. ThunderSTORM: a comprehensive ImageJ plug-in for PALM and STORM data analysis and super-resolution imaging. Available from: https://academic.oup.com/bioinformatics/article/30/16/2389/2748167.
5. F. O. Fahrbach et al., "Propagation stability of self-reconstructing Bessel beams enables contrast-enhanced imaging in thick media," Nature Communications volume 3, Article number: 632 (2012).
6. Lustre. Available from: http://lustre.org/.
7. Adaptive Computing. Torque Resource Manager. Available from: http://www.adaptivecomputing.com/products/open-source/torque/.
8. LibTIFF - TIFF Library and Utilities. Available from: http://www.simplesystems.org/libtiff/.
9. rsync. Available from: https://rsync.samba.org/.
10. BMC Bioinformatics. ImageJ2: ImageJ for the next generation of scientific image data. Available from: https://bmcbioinformatics.biomedcentral.com/articles/10.1186/s12859-017-1934-z.
11. XVFB. Xvfb − virtual framebuffer X server for X Version 11,. Available from: https://www.x.org/releases/X11R7.7/doc/man/man1/Xvfb.1.xhtml.
12. SQLite. Available from: https://www.sqlite.org/index.html.
13. Europe PMC. Fourier ring correlation as a resolution criterion for super-resolution microscopy,. Available from: http://europepmc.org/abstract/MED/23684965.
14. Bitbucket. Torque and Thunderstorm. Available from: https://bitbucket.org/account/user/cbc-group/projects/LOC.
